# Development of *Trypanosoma cruzi in vitro* assays to identify compounds suitable for progression in Chagas’ disease drug discovery

**DOI:** 10.1371/journal.pntd.0006612

**Published:** 2018-07-12

**Authors:** Lorna M. MacLean, John Thomas, Michael D. Lewis, Ignacio Cotillo, David W. Gray, Manu De Rycker

**Affiliations:** 1 Drug Discovery Unit, Wellcome Centre for Anti-Infectives Research, School of Life Sciences, University of Dundee, Dundee, United Kingdom; 2 London School of Hygiene & Tropical Medicine, London, United Kingdom; 3 GlaxoSmithKline, Diseases of the Developing World, Tres Cantos, Madrid, Spain; Instituto de Investigaciones Biotecnológicas, ARGENTINA

## Abstract

Chagas’ disease is responsible for significant mortality and morbidity in Latin America. Current treatments display variable efficacy and have adverse side effects, hence more effective, better tolerated drugs are needed. However, recent efforts have proved unsuccessful with failure of the ergosterol biosynthesis inhibitor posaconazole in phase II clinical trials despite promising *in vitro* and *in vivo* studies. The lack of translation between laboratory experiments and clinical outcome is a major issue for further drug discovery efforts. Our goal was to identify cell-based assays that could differentiate current nitro-aromatic drugs nifurtimox and benznidazole from posaconazole. Using a panel of *T*. *cruzi* strains including the six major lineages (TcI-VI), we found that strain PAH179 (TcV) was markedly less susceptible to posaconazole *in vitro*. Determination of parasite doubling and cycling times as well as EdU labelling experiments all indicate that this lack of sensitivity is due to the slow doubling and cycling time of strain PAH179. This is in accordance with ergosterol biosynthesis inhibition by posaconazole leading to critically low ergosterol levels only after multiple rounds of division, and is further supported by the lack of effect of posaconazole on the non-replicative trypomastigote form. A washout experiment with prolonged posaconazole treatment showed that, even for more rapidly replicating strains, this compound cannot clear all parasites, indicative of a heterogeneous parasite population *in vitro* and potentially the presence of quiescent parasites. Benznidazole in contrast was able to kill all parasites. The work presented here shows clear differentiation between the nitro-aromatic drugs and posaconazole in several assays, and suggests that *in vitro* there may be clinically relevant heterogeneity in the parasite population that can be revealed in long-term washout experiments. Based on these findings we have adjusted our *in vitro* screening cascade so that only the most promising compounds are progressed to *in vivo* experiments.

## Author summary

Chagas’ disease is caused by the protozoan parasite *Trypanosoma cruzi*, and is a major health problem in Latin America. There is an urgent need for new treatments as the few currently used drugs have many serious drawbacks. New potential drugs inhibiting parasite ergosterol biosynthesis have recently failed in the clinic in spite of showing promising results in lab-based cellular models of the disease. To understand why these may have failed we have devised new cellular assays that predict poor performance of ergosterol biosynthesis inhibitors. The new assays comprise of a *T*. *cruzi* strain panel drug efficacy assay that encompasses the six key genetic lineages and a compound washout assay that assesses the ability of candidate drugs to kill all parasites. The ergosterol biosynthesis inhibitor posaconazole performed poorly against the slow replicating/ cycling PAH179 strain and in the washout assay. We conclude that a minimum number of replications are likely to be required for posaconazole to cause a cytocidal effect and not all parasites are susceptible to inhibition of ergosterol biosynthesis. Our new assays will allow early identification of such compounds so that only the most promising drug candidates are progressed towards the clinic.

## Introduction

*Trypanosoma cruzi* is a protozoan parasite (Order Kinetoplastida, Family Trypanosomatidae) responsible for Chagas’ disease, also known as American trypanosomiasis. *T*. *cruzi* parasites are predominantly transmitted to humans as metacyclic trypomastigote forms (non-dividing) in the faeces of infected haematophagous triatomine bugs at the bite site. Entry is either through the wound or transfer to neighbouring mucosa [1]. *T*. *cruzi* transmission can also occur congenitally, via organ transplantation, blood transfusion, or orally by ingestion of parasite contaminated food and drink [2–5]. Chagas’ disease is a zoonotic disease which is endemic in Latin America with an estimated 7–8 million people infected across 21 countries resulting in 12,500 deaths per year, making it one of the leading causes of cardiovascular morbidity and premature death in this region [6, 7]. It has a severe economic impact [8] and is also becoming a global public health problem through human migration [9–14]. Chagas’ disease has two phases, an initial acute phase (typically lasting 2 months) [15], followed by a chronic phase which is life-long. Once the acute phase subsides, patients enter the chronic indeterminate phase. Several years later approximately 30% of people display severe clinical pathologies developing cardiac problems and 10% of cases will develop digestive disorders such as megacolon and megaoesophagous, or neurological dysfunction or a combination of these [16, 17]. In addition, reactivation can occur in patients with the indeterminate chronic form if they become immuno-compromised [18, 19].

*T*. *cruzi* is a highly genetically diverse parasite having diversified from its most recent common ancestor an estimated 3–4 million years ago [20]. *T*. *cruzi* strains have been categorised into six major genetic lineages or discrete typing units (DTU) TcI-VI [21–24]. DTU distribution is associated with geography, ecology and transmission cycle, and it is thought that genetic diversity between strains may be associated with variation in Chagas’ disease pathology and outcome [23, 25]. TcI, II and V cause most human infections. TcI is widely distributed with many silvatic reservoirs and is genetically highly diverse. DTU TcV and VI are hybrid lineages which have originated from recent (within 60,000 years) hybridisation between TcII and TcIII [20]. TcV and VI are almost exclusively found in domestic transmission cycles, particularly in areas where cases of severe Chagas’ disease manifesting with megasyndromes are common [25–27]. TcV is also involved in 80–100% of congenital transmission cases in Argentina, Bolivia, Southern Brazil, Chile and Paraguay [28–32]. On average congenital transmission occurs in 5% of chronically infected mothers [33]. The success of vector control programmes means that congenital transmission is increasingly important, indeed a recent report estimated that 22% of new cases annually were caused by congenital transmission rather than vectorial transmission [34]. Despite this genetic diversity and its epidemiological and likely clinical relevance, a very limited number of strains are used in drug development.

The current drugs available to treat Chagas’ disease are benznidazole (2-nitroimidazole) and nifurtimox (5-nitrofuran) which were developed over four decades ago. These nitroheterocyclic compounds are most effective in the acute phase of disease [35, 36]. However, efficacy varies geographically and there is no consistently effective treatment for established chronic Chagas’ disease [35, 37–40], which is the most prevalent clinical presentation. These drugs can also cause severe side effects and require long treatment duration (60–90 days) which adversely affects patient compliance. The urgent need for new, less toxic drugs led to the identification of ergosterol biosynthesis inhibitors as new potential chemotherapeutics. Triazole derivative posaconazole is a selective inhibitor of the *T*. *cruzi* ergosterol biosynthesis pathway at the C14*α* sterol demethylase (CYP51) level. *T*. *cruzi* requires endogenous sterols for survival and proliferation at all life cycle stages [41]. Posaconazole was shown to be active against *T*. *cruzi in vitro* and *in vivo*, and produced beneficial effects in combination with benznidazole *in vivo* [42–44]. However, these drugs failed in Phase II clinical trials with up to 92% of cases relapsing during the 10–12 month follow-up period (as determined by detection of *T*. *cruzi* DNA in blood by PCR) [45, 46]. A more recent Phase II trial involving asymptomatic carriers also reported high relapse rate for posaconazole (87%), with benznidazole monotherapy giving 13% relapse during the follow-up period with discontinuation of treatment in 32% patients receiving benznidazole alone or in combination with posaconazole due to adverse side effects [47]. The lack of translation of *in vitro* data and *in vivo* model data to clinical outcome is of clear concern. It may be due to differences in *T*. *cruzi* strain response, drug mode of action being dependent on parasite replication, or sub-optimal systemic drug exposure in patients [48]. Indeed there is evidence that response to triazole drug treatment varies between *T*. *cruzi* strains *in vivo* [49, 50]. More recently, strain-specific variation in triazole activity was reported using an *in vitro* intracellular assay where nitroheterocyclic compounds were largely active across TcI-VI and fast acting, while marked variation in potency against *T*. *cruzi* strains was observed for posaconazole [51]. In addition, using a sensitive *in vivo* bioluminescence imaging system to determine *T*. *cruzi* burden in tissues, it was reported that posaconazole has limited effect on both acute and chronic *T*. *cruzi* infections in mice while benznidazole gave 100% sterile cure after 20 days treatment followed by immunosuppression to increase the detection of relapse [52].

To develop future chemotherapy against Chagas’ disease and improve translation to a successful clinical outcome we must ensure robust *in vitro* assays are employed in conjunction with reproducible *in vivo* models that clearly demonstrate sterile cure. To identify new phenotypic hits against *T*. *cruzi* our screening cascade consists of an intracellular image-based *in vitro* assay using *T*. *cruzi* strain Silvio X10/7 subclone A1 (DTU TcI) and Vero host cells, followed by rate of kill and cidality assays [53]. A CYP51 assay is also carried out so only cidal compounds that do not have CYP51 mode of action are progressed to drug metabolism, pharmacokinetic and *in vivo* efficacy studies. The mouse model uses highly sensitive non-invasive bioluminescence imaging to detect transgenic CLBrener expressing the variant firefly luciferase RE9h [54] in BALB/c mice. This methodology provides accurate assessment of parasite burden over time and in specific tissues at endpoints, which is difficult to achieve by PCR analysis. To investigate potential *T*. *cruzi* strain response variation in new drug candidates we have developed a *T*. *cruzi* strain panel of clinically relevant strains encompassing the six major DTUs (TcI-VI) using an *in vitro* image-based intracellular assay. Here we determine *in vitro* efficacy of current nitrohetercyclic drugs nifurtimox and benznidazole, and failed triazole posaconazole against the replicating amastigote form of TcI-VI strains. For comparison these compounds were also tested in a non-replicative trypomastigote bioluminescence assay using strains Silvio X10/7 clone A1 (TcI) and Tulahuen βgal (TcVI). Further assessment of the proliferation status of intracellular parasites remaining after drug treatment was carried out. In addition, an intracellular *T*. *cruzi* washout assay was developed to determine if very low numbers of viable parasites remain after treatment that could help explain failure in the animal model and clinical trials.

## Materials and methods

### *Trypanosoma cruzi in vitro* culture

A panel of *T*. *cruzi* strains originally isolated from humans was established which included one representative from each of the six major DTU’s (Table 1). TcI strain Silvio X10/7 subclone A1 [55]; TcII strain Y [56], TcIII M6241 Clone 6 [57], TcIV ERA Clone 2 [58], TcV PAH179 Clone 5 [59] and TcVI Tula Clone 2 [57]. TcI strain Silvio X10/7 subclone A1 was provided by A. Fairlamb (University of Dundee); TcII strain Y, TcIII M6241 Clone 6, TcV PAH179 Clone 5 and TcVI Tula Clone 2 were all donated by M. Miles (London School of Hygiene and Tropical Medicine, LSHTM, UK); TcIV ERA Clone 2 was also obtained from M. Miles LSHTM with agreement of Hernan Carrasco (Universidad Central de Venezuela); CLBrener expressing pTRIX2-PpyRE9h red-shifted was obtained from J. Kelly (LSHTM, UK).

**Table 1:** *T*. *cruz*i strain panel encompassing the six major *T*. *cruzi* discrete typing units (DTU’s) TcI-VI.

DTU	*T*. *cruzi* Strain ID	Host	Date	Origin
Tc I	Silvio X10/7 Clone A1	Human	1979	Pará, Brazil
Tc II	Y	Human	1953	São Paulo, Brazil
Tc III	M6241 Clone 6	Human	1988	Pará, Brazil
Tc IV	ERA Clone 2	Human	1999	Anzoátegui, Venezuela
Tc V	PAH179 Clone 5	Human	2000	Chaco, Argentina
Tc VI	Tula Clone 2	Human	1988	Tulahuén, Chile
Tc VI	CLBrener	Triatoma Infestans	1963	Rio Grande do Sul, Brazil

Henceforth, these strains will be referred to by name without the clone information. In addition, our animal model strain CLBrener [60] expressing pTRIX2-PpyRE9h red-shifted luciferase [54] was also included in the panel (CLBrenerLuc, TcVI). Tulahuen strain parasites (TcVI) stably expressing β-galactosidase (Tulahuen βgal) [61] were not included in the *T*. *cruzi* strain panel but were used in the trypomastigote assay. These were kindly provided by F. Buckner (University of Washington, Seattle, USA).

All *T*. *cruzi* strains described above, with the exception of the Tulahuen βgal strain, were maintained as epimastigotes *in vitro* at 28°C in RTH/ FCS culture medium (RPMI1640 supplemented with 0.4% trypticase peptone, 0.017 M hepes, 25 μM haemin,10% heat inactivated FCS (GE Healthcare) [62, 63]. Epimastigotes (10^6^ ml^-1^) were inoculated into RTH/ FCS and grown until a high proportion of metacyclic trypomastigote parasites were observed (~7–10 days). Trypomastigote-rich cultures were incubated with a monolayer of Vero cells (African green monkey kidney cells, ECCAC 84113001) at a multiplicity of infection (MOI) of 10–15 overnight at 37°C 5% CO_2_ in Dulbecco’s modified Eagles’s medium (DMEM 4.5 gL^-1^ Glucose & L-Glutamine) (Lonza) supplemented with 10% FCS (GE Healthcare). Extracellular parasites were removed the following day by aspirating cell culture media, washing the Vero cell monolayer with serum-free DMEM three times, followed by addition of fresh complete DMEM. *T*. *cruzi* infected Vero flasks were maintained at 37°C 5% CO_2_, DMEM/ FCS replaced every 48h until trypomastigotes emerged from Vero cells to be used to set-up new Vero cell infections. The routine cycling time through Vero cells varied considerably between strains from 3 to 12 days, therefore, for more slowly cycling strains (PAH179 & Tula), infected Vero cells were pre-incubated for 3 days prior to assay plating to coordinate parasite cycling times through Vero cells.

Tulahuen βgal parasites were maintained in culture by weekly infection of LLC-MK2 cells (Rhesus macaque kidney epithelial cells) in DMEM (Life-Technologies) supplemented with 2% FBS (Biowest, USA) and 100 Uml^-1^ penicillin and 100 μgml^-1^ streptomycin (Sigma-Aldrich). Trypomastigote forms were obtained from the supernatants of LLC-MK2 infected cultures harvested between days 5 and 9 of infection.

### Host cell culture

Vero cells were maintained at 37°C 5% CO_2_ in DMEM (Lonza) supplemented with 10% FCS as described above. Vero cells were sub-cultured every 2 days at a ratio of 1:5 after 5 min treatment with Trypsin-EDTA (Gibco).

LLC-MK2 cells were cultivated in DMEM (Life-Technologies) supplemented with 10% FCS (Biowest, USA), 100 Uml^-1^ penicillin and 100 μgml^-1^ streptomycin (Sigma-Aldrich) at 37°C, 5% CO_2_ and >95% humidity. This cell line was maintained twice a week at a ratio of 1:10.

### *Leishmania donovani in vitro* culture

*Leishmania donovani* strain MHOM/ET/67/HU3:LV9 originally isolated from a patient in Ethiopia 1967 were maintained as promastigotes at 28°C in RPMI1640 (Sigma, Dutch modification) supplemented with 20% FCS (Hyclone), 100 μM adenine, 20 mM MES hydrate, 5 μM haemin, 6-biopterin 3 μM, biotin 1 μM, 1% penicillin and streptomycin. Parasites were established at 10^5^ ml^-1^ and sub-cultured every 3 days.

### *T*. *cruzi* strain genotyping

Total genomic DNA was isolated from *T*. *cruzi* epimastigote or trypomastigote cultures using Qiagen DNAeasy tissue & blood kit according to manufacturer’s instructions.

#### Single-locus typing assay

*T*. *cruzi TcSC5D* gene encodes a putative C-5 sterol desaturase (TcCLB.473111.10, TcCLB.507853.10), this was amplified by PCR followed by direct sequencing to determine DTU-specific genotypes using eight key discriminating single nucleotide polymorphisms (SNP’s) as previously described [64]. Briefly, a 832bp *TcSC5D* fragment was amplified under the following PCR conditions: 50 pmol *TcSC5D*-forward (5´-GGACGTGGCGTTTGATTTAT-3´) & reverse primers (5´-TCCCATCTTCTTCGTTGACT-3´), 100 ng genomic DNA, Platinum PCR Supermix High Fidelity (ThermoFisher Scientific) in a final volume of 50 μl. Samples were denatured at 94°C for 5 min, followed by 35 cycles at 94°C 30 s, 58°C 30 s, 72°C 30 s, and extension at 72°C for 5 min. Amplification products were visualised on 1.2% agarose with ethidium bromide as a single 832 bp product, and subsequently sequenced with the same PCR primers using Applied Biosystems 3730 DNA Analyser. Chromatograms were analysed using Chromas software and alignments generated using Clustal Omega to identify nucleotides at key position 138, 168, 336, 495, 618, 648, 657 and 747 and determine DTU assignment TcI-VI (S1 Table).

#### Triple assay

DTU assignment was made based on genotypes for three PCR-based assays: the amplicon size polymorphism of the *24S rRNA* locus [65] and two restriction fragment length polymorphism assays based on SNPs in the *HSP60* & *GPI* loci [66]. The combined genotypes were used to infer the DTU according to the scheme described by Lewis et al [67].

Amplification reactions contained 0.2 mM of each dNTP, 1.5 mM MgCl_2_, 1 pmol μl^-1^ of each primer, 1 Unit of Taq DNA polymerase (Promega, UK) and approximately 10 ng gDNA. For the *24S rDNA*, PCR primers D71 (5’-AAGGTGCGTCGACAGTGTGG-3’) and D72 (5’-TTTTCAGAATGGCCGAACAGT-3’) were used and conditions comprised an initial denaturation step of 94°C for 3 min then 27 amplification cycles (94°C for 1 min, 60°C for 1 min, 72°C for 1 min) followed by a final elongation step at 72°C for 5 min. Amplicon sizes were visualised on 1X Tris-acetate-EDTA (TAE), 3.5% NuSieve 3:1 agarose (Lonza) gels.

For *GPI* and *HSP60*, PCR amplification conditions comprised an initial denaturation step of 3 min at 94°C followed by 4 cycles (94°C for 30 s, 64°C for 30 s, 72°C for 1 min) followed by 28 cycles (94°C for 30 s, 60°C for 30 s, 72°C for 1 min) and then a final elongation step at 72°C for 10 min. *GPI* primers were 5’-GGCATGTGAAGCTTTGAGGCCTTTTTCAG-3’ (Fwd) and 5’-TGTAAGGGCCCAGTGAGAGCGTTCGTTGAATAGC-3’ (Rev). *HSP60* primers were 5’-GTGGTATGGGTGACATGTAC-3’ (Fwd) and 5’-CGAGCAGCAGAGCGAAACAT-3’ (Rev). For restriction enzyme digestion, 10 μl of PCR product (typically ~1 μg) was digested with 0.25 U μl^-1^ of either *Hha*I for *GPI* or *EcoRV* for *HSP60* for 4 h at 37°C. Restriction fragment sizes were visualised on 1X TAE, 2% agarose gels.

### Compound handling

Compounds included current treatments for Chagas’ disease benznidazole and nifurtimox (both from Sigma), and CYP51 inhibitor posaconazole (Sequoia Research Products). Compound (250 nl in DMSO) was dispensed using LabCyte ECHO into each well (Corning black flat bottomed 384-well plates for the intracellular amastigote assay; Greiner white 384-plates for the trypomastigote assay). Ten-point potency curves were generated (one in three dilution), with technical duplicates for the intracellular assay and in triplicate for the trypomastigote assay. All potency determinations were performed a minimum of 3 independent times. A top concentration of 50 μM was used for benznidazole and nifurtimox, and 1 μM for posaconazole in the intracellular assay (all wells contained 0.5% DMSO), while a top concentration of 50 μM was used for all three drugs in the trypomastigote assay. Drug potencies are reported as pEC50 which is–Log (EC_50_ [M]).

### *T*. *cruzi* intracellular assay

Silvio X10/7, Y, M6241, ERA and CLBrenerLuc trypomastigotes were incubated at 37°C 5% CO_2_ with 1.6×10^7^ Vero cells overnight at MOI10 in a T225 flask. Extracellular parasites were removed by aspirating cell culture supernatant, and washing Vero monolayer with 10 ml serum-free DMEM three times. Vero cells were then harvested by trypsinization and dispensed at 2×10^3^ per well (50 μl in DMEM/ 1% FCS) into 384 well plates pre-stamped with drug in DMSO using automated washer/ dispenser EL406 and liquid handling software (Biotek). Plates were then incubated at 37°C in 5% CO_2_ for 72, 96 and 120 h.

Plates were subsequently fixed with 4% formaldehyde for 20 min, permeabilised and stained with 5 μgml^-1^ Hoechst 33342/ 0.1% Triton/ PBS Thimerosal 20 min. Automated imaging was performed by the Operetta high content imaging system using 20x objective (PerkinElmer). Images were analysed with an algorithm generated in Columbus (PerkinElmer) to segment Vero cell nuclei, Vero cell cytoplasm and parasite nuclei/ kinetoplasts, reporting number of amastigotes per Vero cell and total number of Vero cells. Compound potencies against *T*. *cruzi* parasites were calculated in IDBS Activitybase using total number of amastigotes per well and Vero toxicity curves generated using number of host cells. All data was normalised to percent inhibition based on the raw data values for the 100% effect control (50 μM nifurtimox) and the 0% effect control (DMSO) on each plate at each time point. Curve fitting was carried out using a four-parameter equation as previously described [68].

### Parasite replication

To determine amastigote replication rates for each strain, the *T*. *cruzi* intracellular assay was established as described above with identical plates set-up for each strain and fixed daily to determine number of amastigotes from Day 0 (day of plating) up to Day 5. *T*. *cruzi* amastigote doubling time (DT) was determined by fitting the 0–72 h growth data to exponential expression N = N_0_.e^Ke.t^ (N = number of cells at time t, N_0_ = number of cells at t = 0 h, Ke is the exponential constant). DT was calculated as ln(2)/Ke. There are 32 technical replicates for each time point per plate, and at least 3 biological replicates for each strain.

### *T*. *cruzi* trypomastigote assays

Silvio X10/7 trypomastigotes were harvested from infected Vero cells in a T225 flask infection set-up at MOI 5 for 5 days. Trypomastigotes (5×10^4^ per well) in 50 μl of DMEM/ 10% FCS were dispensed into white 384 plates (Greiner) pre-stamped with 250 nl compound. Plates were incubated at 37°C in 5% CO_2_ for 24, 48 and 72 h. Cell Titer-Glo cell viability solution (Promega) was added to each well (50 μl) incubated at room temperature for 10 min and luminescence read by Envision plate reader (Perkin Elmer). This assay quantifies the amount of ATP present which is directly proportional to the number of metabolically active cells. All data was normalised as described above (100% effect control 50 μM nifurtimox, 0% effect control DMSO).

Tulahuen βgal trypomastigotes were harvested from LLC-MK2 host cells after 5–9 days infection. Trypomastigotes (5×10^4^ per well) in 50 μl of DMEM/ 2% FCS/ 100 Uml^-1^ penicillin and 100 μgml^-1^ streptomycin were dispensed and processed as described above.

### Cell proliferation, antibody labelling and imaging

Proliferation of Silvio X10/7 trypomastigotes was measured using Click-iT Plus EdU AlexaFluor 488 Imaging Kit (ThermoFisher Sci). EdU (5-ethynyl-2-deoxyuridine) is a nucleoside analog of thymidine that is incorporated into newly synthesised DNA. Detection is via a copper catalysed covalent click reaction between picolyl azide which is contained in the EdU and an alkyne contained in the AlexaFluor 488 label. Silvio X10/7 trypomastigotes (2×10^7^) were harvested from Vero cells, washed with PBS, labelled live with 10 μM EdU labelling solution for 2 h at 37°C 5% CO_2_. *L*. *donovani* LV9 mid-log promastigotes were included as a replicating cell control and incubated at 28°C 2 h. After media aspiration, cells were fixed in 4% formaldehyde/PBS for 20 min, washed three times with 3% fatty acid free BSA blocking solution (BB International), permeabilised with 0.5% Triton X-100 20 min, washed with blocking solution, and incubated with 100 μl of Click-iT Plus reaction cocktail for 30 min in the dark. Cells were washed twice and incubated in blocking reagent overnight at 4°C. Edu labelled cells were also labelled with *L*. *major* rabbit polyclonal anti-PFR1 (paraflagellar rod) antibody [69] 1:200 in blocking solution for 1 h. Parasites were washed in block, incubated with goat anti-rabbit IgG AlexaFluor-647 for secondary detection of anti-PFR 1 h (1:200; ThermoFisher Sci), washed in PBS and resuspended in 5 μgml^-1^ Hoechst 33342 (DNA stain) 20 min. Parasites were subsequently washed in PBS, resuspended in 100 μl PBS and allowed to adhere to poly-D-lysine coated 96 well Cell Carrier black plates (Perkin Elmer) for 20 min before imaging with the Operetta high content microscope 40XHi NA objective. EdU (Ex/ Em 495/ 519 nm), PFR/ AlexaFluor 647 (Ex/ Em 650/ 670 nm), Hoechst (Ex/ Em 350/ 461 nm) plus bright field, ten 1 μm slice stack.

Silvio X10/7 and PAH179 infected Vero cells (MOI10) were also labelled with EdU and Hoechst as described above. However, labelling was performed on infected Vero cells which were already adherent on 96 well black Cellbind plates (Corning) seeded at 4×10^3^ and 10^3^ per well after 5 days treatment with 1 μM posaconazole, 50 μM benznidazole, 5 μM nifurtimox or 0.5% DMSO. Control wells without EdU labelling were also set-up for each condition and were processed the same way but without incubation with EdU labelling solution. Plates were imaged using the Operetta high content microscope with 40X objective for EdU, Hoechst and bright field as above, five 1 μm slice stack, 16 fields/well. An algorithm was generated in Columbus to determine percent infected cells, percent EdU positive Vero and EdU positive intracellular parasites. For publication purposes deconvolution images were acquired.

### *T*. *cruzi* washout assay

Vero cells (1.6 ×10^7^) were infected at MOI 5 with Silvio X10/7 trypomastigotes in MEM media (Gibco) containing 10% FCS (Hyclone) followed by washing of the monolayer to remove any extracellular trypomastigotes. Total Vero infection time with Silvio X10/7 trypomastigotes prior to compound treatment was either 40 h or 16 h. Infected cells were trypsinised and plated in T25 flasks (1.8×10^6^ cells per flask, MEM, 1% FCS) or incubated for a further 24 h prior to trypsinisation and plating. Infected cells were incubated with compounds at the indicated concentrations for either 8 days or 16 days. Every 4 days the media was replaced with fresh compound-containing media. After the treatment period, monolayers were washed extensively. The cultures were then maintained, replacing media twice weekly, until trypomastigotes could be observed by light microscopy. Observations were carried out twice weekly for up to 60 days post washout.

## Results

### *T*. *cruzi* strain genotyping

All *T*. *cruzi* strains included in this study were confirmed to be the expected DTU by either the triple assay (PCR product size polymorphism of the *24S rRNA* locus; PCR-RFLP based on SNP’s in the *GPI* and *HSP60* loci) and/ or the single assay (DTU discriminating SNP’s in *T*. *cruzi TcSC5D* gene) (S2 Table, S1 Fig).

### *T*. *cruzi* amastigote growth rate & level of Vero cell infection varies between strains

Growth of intracellular amastigotes varies between *T*. *cruzi* strains. Silvio X10/7, M6241, Tula and CLBrenerLuc strains demonstrated the fastest doubling times and higher starting (day 0) percent infected Vero cells. Silvio X10/7 and M6241 gave similar Vero cycling times (4–5 days between infection and egress of parasites) while CLBrenerLuc and Tula cycled every 6–8 d (Table 2). Y, ERA and PAH179 strains displayed the slowest doubling times (24 to 26 h). However, Vero cycling times varied considerably with Y and ERA cycling rapidly (every 3–4 days), while PAH179 had the slowest cycling time (8–12 days). The duration of our established primary *T*. *cruzi* screening assay using Silvio X10/7 infected Vero cells is 72 h. To improve assessment of compounds that may have modes of action that are replication dependent, the *T*. *cruzi* strain panel assay was extended to 120 h post-infection (Table 2). At this time point Y strain trypomastigotes had emerged and some re-infection was observed. Although there was some variation in doubling time between biological replicates, strain PAH179 achieved the lowest number of divisions at 120 h and reached stationary growth phase at lower total amastigote counts than other strains (Table 2, S2 Fig). Strains Y and PAH179 gave the lowest starting percent infected Vero (day 0). Despite differences between strains in infection level and doubling times the total number of amastigotes counted at the assay end point (120 h) was always more than 10^3^ per well for all *T*. *cruzi* panel strains providing a sufficient window between the 0% (DMSO) and 100% (50 μM nifurtimox) effect controls to generate potency curves.

**Table 2:** *T*. *cruz*i strain panel amastigote doubling time (DT), average ±SD, calculated using 0-72h growth curve data [see Methods] for at least 3 biological replicates. This value was used to calculate the number of divisions in 72 h. Number of divisions in 120 h were calculated from DT determined using number of amastigotes at 0 and 120 h. Cycling time defines the time in days between the start of Vero cell infection with *T*. *cruzi* tissue culture derived trypomastigotes and the emergence of trypomastigote parasites. Level of parasite infection (Day 0) for each strain represented as percent infected Vero cells (average± SD).

	Doubling Time (h)	Divisions/ 72 h	Divisions / 120 h	Cycling times (d)	% infected Vero
Silvio X10/7	19.6 ± 1.5	3.7 ± 0.3	4.6 ± 1.3	4–5	31 ± 15
Y	25.3 ± 5.8	3.0 ± 0.7	3.5 ± 1.7	3	11 ± 10
M6241	18.9 ± 1.6	3.8 ± 0.3	4.3 ± 0.7	4–5	25 ± 15
ERA	24.3 ± 4.8	3.0 ± 0.6	3.9 ± 0.8	4	30 ± 16
PAH179	25.6 ± 7.4	3.0 ± 0.8	3.3 ± 1.2	8–12	16 ± 11
Tula	19.5 ± 2.9	3.7 ± 0.5	5.7 ± 1.8	7–8	23 ± 19
CLBrenerLuc	19.5 ± 2.8	3.7 ± 0.6	3.9 ± 1.3	6–7	32 ± 21

### Intracellular assay—Posaconazole shows poor activity against slowly replicating/ cycling *T*. *cruzi*

**Fig 1:**
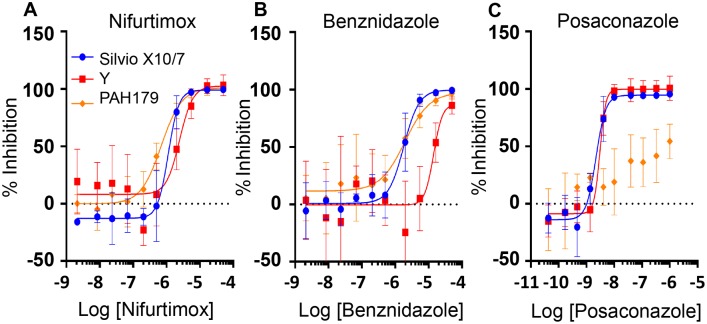
Drug efficacy against *T*. *cruzi* intracellular amastigotes. Strains Silvio X10/7, Y and PAH1790 assessed after 5 days treatment with nifurtimox **(A)** benznidazole **(B)** and posaconazole **(C)**. (Representative dose-response curves of at least 3 biological replicates normalised to 0% effect DMSO and 100% effect 50 μM nifurtimox controls. Average±SD). The x axis is the Log of the compound concentration in Molar.

Nifurtimox was active across all seven *T*. *cruzi* strains from six major DTUs with a maximum six-fold difference in potency observed between strains (CLBrenerLuc v Y) at 120 h (Table 3; Fig 1A illustrates nifurtimox dose response data for three key DTU strains). Benznidazole showed good activity across five *T*. *cruzi* strains with a maximum inhibition of at least 99% relative to nifurtimox at 120 h (Table 3). However, reduced efficacy against strain Y was observed. Benznidazole was 5–13 fold less potent against strain Y than other strains at 120 h reaching 90% maximum inhibition (Table 3, Fig 1B).

**Table 3:** Drug efficacy against *T*. *cruzi* panel strains at 120 h. pEC_50_ = –Log (EC_50_ [M]), average of at least three biological replicates ± SD. Additional data for 72 h and 96 h time points available in S3 and S4 Tables respectively.

*T*. *cruzi* Strain ID	Nifurtimox		Benznidazole		Posaconazole	
	pEC_50_	Max inhibition (%)	pEC_50_	Max inhibition (%)	pEC_50_	Max inhibition (%)
Silvio X10/7	6.1 ± 0.2	100 ± 1	5.7 ± 0.2	100 ± 2	8.6 ± 0.1	97 ± 3
Y	5.7 ± 0.2	103 ± 5	4.8 ± 0.1	90 ± 4	8.6 ± 0.2	108 ± 10
M6241	6.0 ± 0.1	105 ± 5	5.5 ± 0.2	105 ± 5	8.7 ± 0.3	104 ± 6
ERA	6.3 ± 0.0	103 ± 3	5.9 ± 0.1	103 ± 3	8.9 ± 0.1	91 ± 9
PAH179	6.3 ± 0.2	102 ± 5	5.7 ± 0.1	102 ± 5	<6.0	65 ± 14
Tula	6.3 ± 0.4	100 ± 1	5.9 ± 0.1	99 ± 2	8.4 ± 0.1	94 ± 5
CLBrener Luc	6.5 ± 0.1	100 ± 2	5.9 ± 0.2	101 ± 2	8.5 ± 0.2	94 ± 6

**Fig 2:**
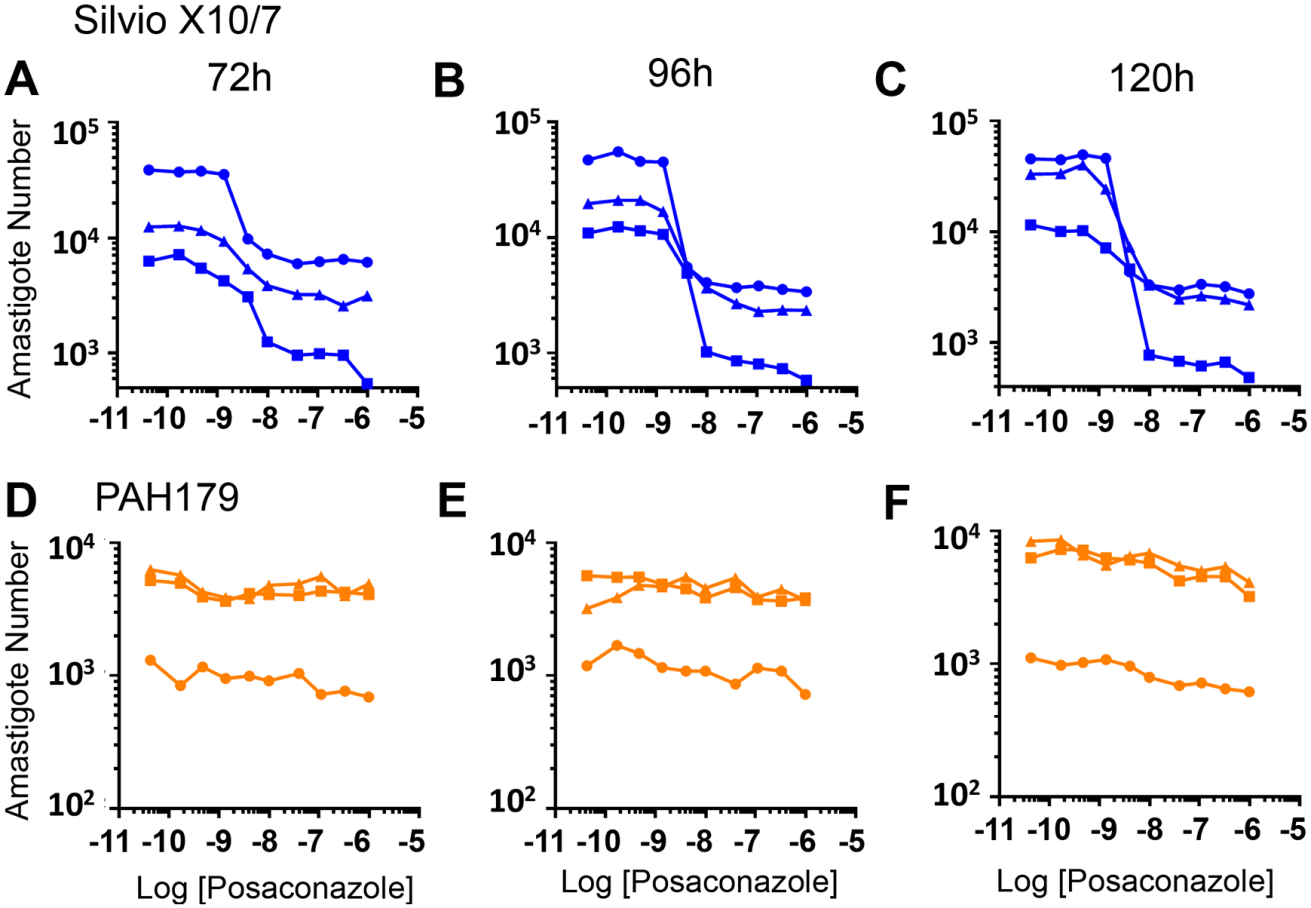
Raw dose-response data for *T*. *cruzi* intracellular amastigotes after treatment with posaconazole at 72 h, 96 h and 120 h for strains Silvio X10/7 (A-C) and PAH179 (D-F) (3 biological replicates shown). The x axis is the Log of the compound concentration in Molar.

The triazole posaconazole, was active against five *T*. *cruzi* strains at low nanomolar concentrations at 120 h (Table 3, Fig 1C). However, posaconazole was more slowly acting than the nitroheterocyclic compounds tested, with clear differences between the strains. While >90% inhibition was observed for Silvio X10/7, Y, and M6241 at 72 h, ERA, Tula and CLBrenerLuc showed <75% inhibition (S3 Table). By 120 h >90% inhibition was observed against these strains (Table 3, Fig 1C). Interestingly, posaconazole showed only minimal activity against strain PAH179. Intracellular *T*. *cruzi* inhibition was only 17% at 72 h, reaching a maximum of 65% at 120 h (Tables S3 & 3, Fig 1C). After 72 h and 96 h treatment there was little discernible effect of posaconazole on intracellular PAH179 parasite counts while at 120 h parasite numbers started to decrease at high compound concentrations but not to the extent observed for other strains (Fig 2). (S3 Fig illustrates non-normalised dose-response data for nifurtimox, benznidazole and posaconazole at 120h for all strains). This poor activity against the strain that replicates and cycles most slowly suggests posaconazole mode of action is replication dependent, possibly requiring completion of several replication cycles for full activity.

### Trypomastigote assay—Posaconazole has minimal activity against trypomastigote stage parasites

**Fig 3:**
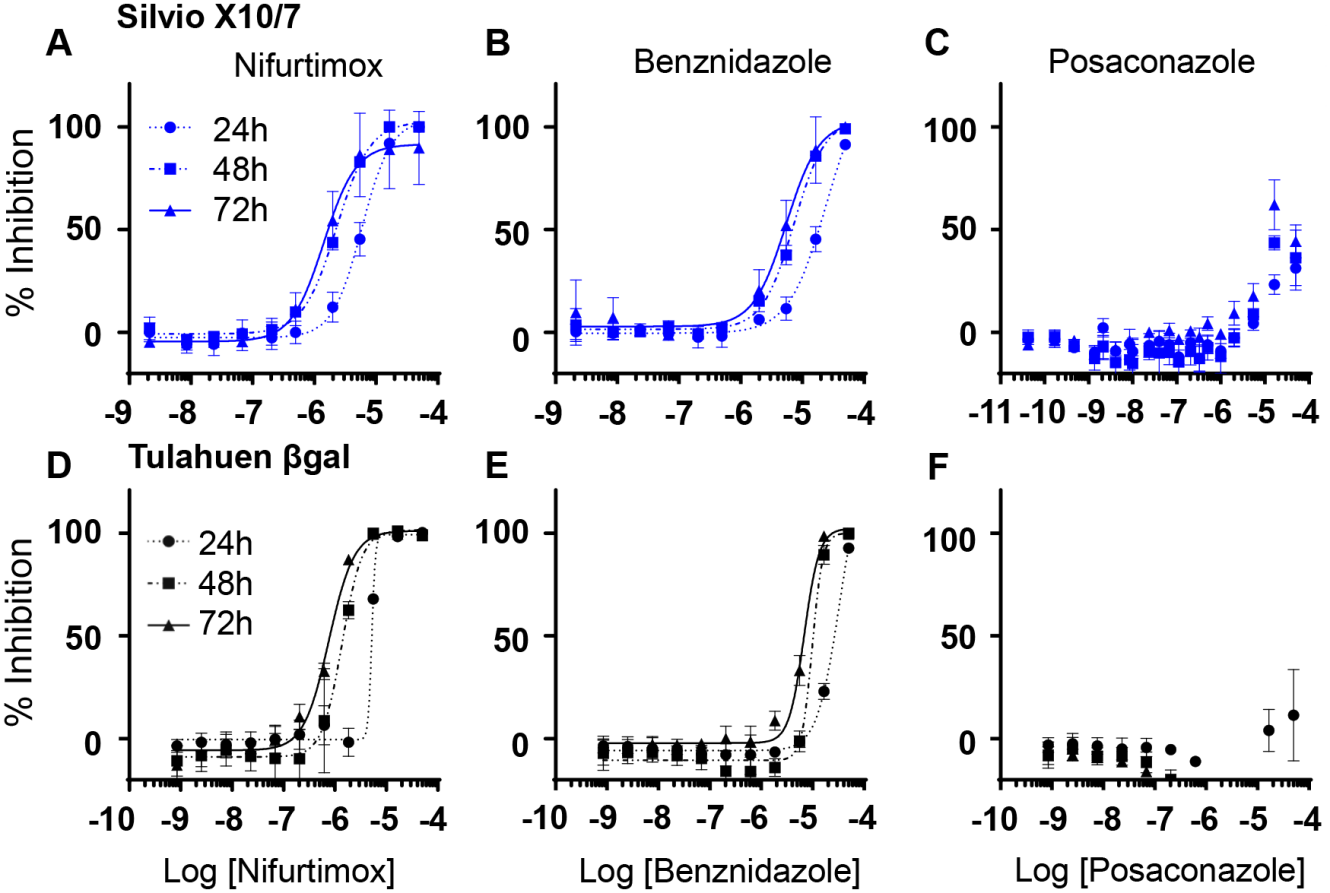
Drug efficacy against *T*. *cruzi* trypomastigotes. Silvio X10/7 **(A-C)** and Tulahuen βgal **(D-F)** trypomastigotes after 24, 48 and 72 h treatment with nifurtimox (**A & D),** benznidazole (**B & E)** and posaconazole (**C & F).** (Dose-response curves of three biological replicates. Average±SD). The x axis is the Log of the concentration of the drug in Molar.

Nifurtimox and benznidazole were both active against Silvio X10/7 (TcI) and Tulahuen βgal (TcVI) trypomastigote parasites (non-replicating stage) (Table 4, Fig 3A, 3B, 3D and 3E). However, posaconazole was not active against Tulahuen βgal trypomastigotes and poorly active against Silvio X10/7 at 72 h (Table 4, Fig 3C and 3F), with a very large drop-off in potency (> 3000-fold at 72 h) between the intracellular and trypomastigote assays, again suggesting posaconazole efficacy is replication dependent.

**Table 4:** Efficacy of nifurtimox, benznidazole and posaconazole against *T*. *cruzi* Silvio X10/7 and Tulahuen βgal trypomastigotes at 72 h. pEC_50_ = –Log (EC_50_ [M]), average of at least three biological replicates ± SD. Additional data for 24 h and 48 h time points available in S5 Table.

*T*. *cruzi* Strain ID	Nifurtimox		Benznidazole		Posaconazole	
	pEC_50_	Max inhibition (%)	pEC_50_	Max inhibition (%)	pEC_50_	Max inhibition (%)
Silvio X10/7	5.8 ± 0.1	100 ± 0	5.4 ± 0.0	100 ± 0	4.7 ± 0.2	70 ± 4
Tulahuen βgal	6.1 ± 0.0	100 ± 1	5.2 ± 0.0	100 ± 1	<4.3	-5 ± 2

### EdU labelling- Non-replicating trypomastigotes and replicating amastigote stage PAH179 strain parasites remain after posaconazole treatment

The above data are in agreement with posaconazole being inactive against parasites that do not go through a sufficient number of cell divisions. To determine if PAH179 parasites remaining after posaconazole treatment are non-replicating forms we assessed the effect of posaconazole on the fraction of replicating parasites using EdU (nucleoside analog of thymidine) labelling to identify cells in S-phase of growth. As a positive proliferative cell control, EdU labelling of *L*. *donovani* LV9 mid-log promastigotes was performed where EdU staining was observed in 54% of LV9 (118 cells analysed) co-localising with the parasite nucleus (S4A Fig). This is a non-synchronous population but it also cannot be ruled out that not all replicating cells are detected under these labelling conditions. As expected, no EdU labelling was detected in Silvio X10/7 trypomastigotes (161 cells analysed) which are a non-replicating developmental form (S4C Fig). Presence of a flagellum was detected by PFR1 labelling in both LV9 and Silvio X10/7 confirming Silvio X10/7 trypomastigote morphology in conjunction with Hoechst staining revealing characteristic positioning of nuclei and kinetoplasts (S4C Fig).

**Fig 4:**
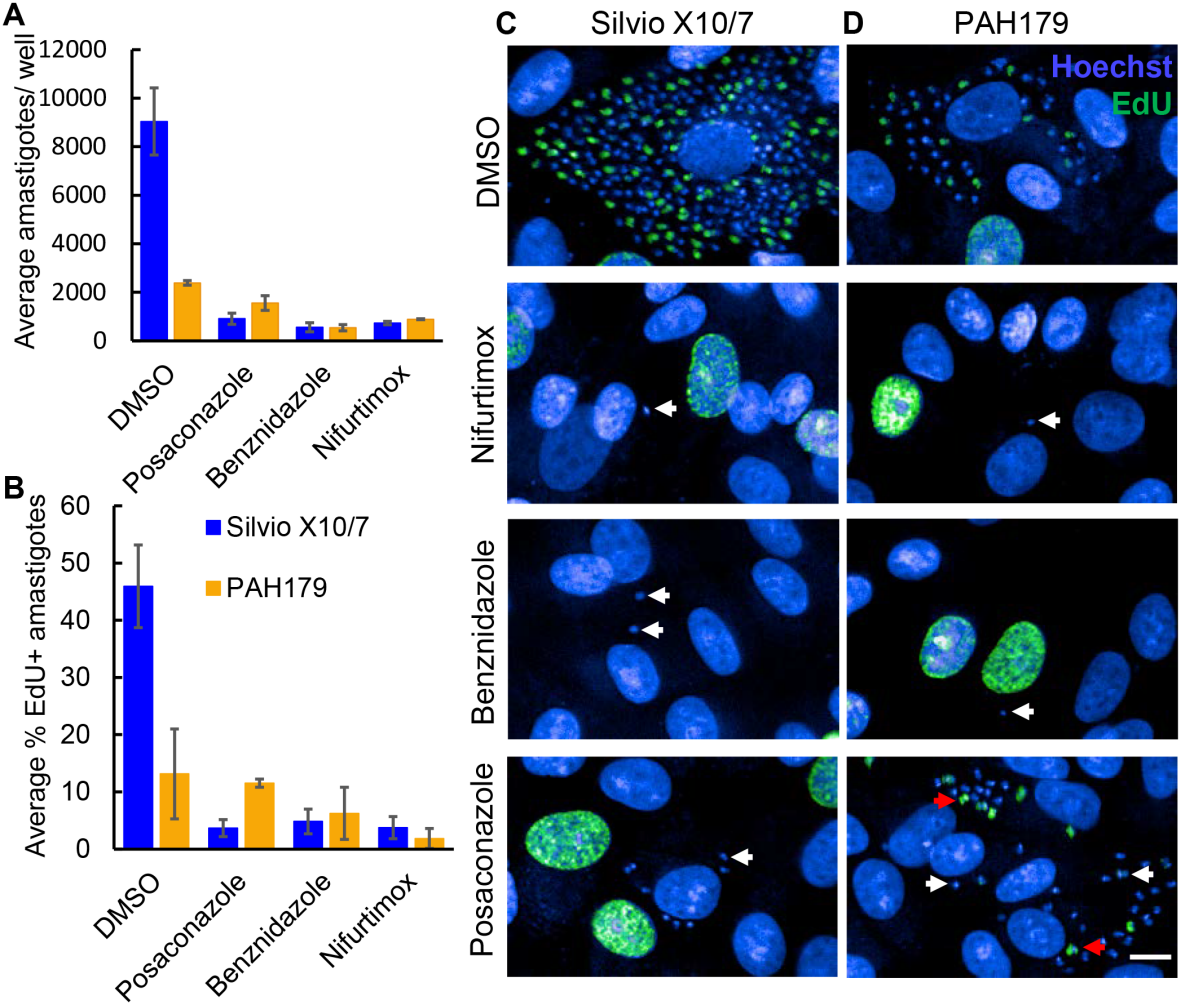
Detection of cells in S phase growth by EdU labelling of intracellular *T*. *cruzi* strains Silvio X10/7 and PAH179. Number of amastigotes per well (average ±SD) determined by Hoechst staining after 5 days treatment with DMSO, 5 μM nifurtimox, 50 μM benznidazole and 1 μM posaconazole **(A)**. Percent EdU positive amastigotes **(B)** after treatments described above using the automated image analysis algorithm. Imaging by HTS microscopy of Silvio X10/7 **(C)** and PAH179 **(D)** infected Vero labelled with EdU AlexaFluor 488 and Hoechst, treated for 5 days with DMSO, 5 μM nifurtimox, 50 μM benznidazole and 1 μM posaconazole. Parasites remaining after treatment are highlighted by white arrows (Hoechst), replicating parasites remaining after treatment are highlighted by red arrows (EdU AlexaFluor488). Bar 10μm.

EdU labelling of intracellular Silvio X10/7 and PAH179 was subsequently performed. After 5 days incubation without drugs (0.5% DMSO, vehicle control) 46% of Silvio X10/7 and 13% of PAH179 amastigotes were EdU positive (Fig 4B). After 5 days treatment with 5 μM nifurtimox, 50 μM benznidazole, or 1 μM posaconazole, a significant reduction in total number of Silvio X10/7 amastigotes and EdU positive amastigotes was observed (Fig 4A and 4B Anova p<0.001). These drug concentrations were chosen as they show good activity against Silvio X10/7 but are not toxic to Vero cells. Of the Silvio X10/7 amastigotes remaining after each treatment only 4–5% of remaining parasites were categorised as EdU labelled. However, visual inspection of the images showed that the majority of remaining parasites were EdU false positive detections by the image analysis algorithm (Fig 4C bottom three panels**)**. After 5 days 5 μM nifurtimox, or 50 μM benznidazole treatment, a significant reduction in total intracellular PAH179 parasites was also observed (Anova P<0.005) with only 2–6% EdU positive (Fig 4A and 4B). Again, upon image visualisation, no EdU positive remaining parasites were observed (Fig 4D middle two panels). However, 5 days 1 μM posaconazole treatment only cleared half of the intracellular parasites, and 12% of the remaining parasites were EdU positive (Fig 4A and 4B) which is a similar proportion as DMSO treatment (13%). Thus, posaconazole is not active against non-replicating intracellular PAH179 strain parasites and replicating PAH179 can be refractory to killing by posaconazole, consistent with parasite death only after several replication cycles (Fig 4D bottom panel). Although it is possible that slowly replicating parasites would not be detected under these EdU labelling conditions, this method does detect replicating *T*. *cruzi*. Individual images for each strain are shown in S5 and S6 Figs. Infected Vero control wells not incubated with EdU show no background staining (S7 and S8 Figs). The proportion of EdU labelled Vero cells did not significantly change with any of the drug treatments (Anova p>0.05) (S9 Fig).

### *T*. *cruzi* washout assay—Benznidazole is curative *in vitro* and posaconazole is not

The experiments above do not demonstrate that the nitroheterocyclic compounds can kill all parasites, neither do they exclude that longer treatment with posaconazole could be efficacious. To address these questions a washout experiment was performed using Silvio X10/7 infected Vero cells treated for either 8 or 16 days with high concentrations of benznidazole or posaconazole (12.5–50 times EC_50_). Treated cells were then maintained for up to 60 days and culture flasks were assessed for presence of trypomastigotes. Lack of recrudescence within 60 days was considered evidence for complete inactivation of all parasites. Recrudescence of parasites was seen for both compounds with the 8 day treatment regimen, but emergence of trypomastigotes was significantly delayed with benznidazole compared to posaconazole (benznidazole relapse: 14 to 21 days after washout, posaconazole: 3 days after washout) (Table 5). After 16 days of treatment no parasite recrudescence was seen with benznidazole for over 60 days, suggesting it is able to kill all parasites *in vitro*. Posaconazole treated cells did relapse, with parasites emerging after only 11 days.

**Table 5:** *T*. *cruzi* washout experiment with benznidazole and posaconazole treated Silvio X10/7 infected Vero cells. Regimen indicates the number of continuous days the cells were treated with each concentration. Compounds were replaced every 4 days. Relapse day was the first day trypomastigotes can be observed by light microscopy in culture after washout, negative numbers indicate that trypomastigotes were observed prior to washout.

Compound	DMSO		Benznidazole					Posaconazole				
**Regimen**	8 d	16 d	8 d			16 d		8 d			16 d	
**Concentration (fold EC**_**50**_**)**	0		12.5	25	50	25	50	12.5	25	50	50	
**Relapse Day**	-4	-12	14	17	21	>60	>60	3	3	3	11	11

## Discussion

Here we describe development of a *T*. *cruzi* strain panel intracellular assay and a washout assay to test the ability of compounds to show activity across the six main *T*. *cruzi* DTU’s and achieve sterile cure *in vitro*. The purpose of this work is to assess if these assays could improve translation from *in vitro* cellular assays to *in vivo* efficacy.

In the strain panel we observed variation in both doubling time and host cell cycling time between strains, with PAH179 (TcV) displaying both the slowest replication rate and longest cycling time. The reported amastigote doubling times are estimates using day 0 to day 3 counts. The actual replication rates may be higher than we report for fast cycling parasites such as the Y strain which cycles every 3 days as extracelluar trypomastigotes are removed by washing before staining and counting. In addition, growth rate and cycling time of some strains increased over time in culture. However, the PAH179 strain continued to be the slowest cycling strain with a minimum of 8 days before trypomastigotes emerged.

Interestingly we observed strain-dependent differences for the drugs tested. In general the nitroheterocyclic compounds nifurtimox and benznidazole were active across strains, with a lower level of potency against the Y strain (TcII), especially for benznidazole, whereas posaconazole was markedly less active against PAH179 (TcV), suggesting that replication and cycling times may affect posaconazole efficacy. This is supported by the very poor activity of posaconazole against non-replicative trypomastigotes. Our results align with the data generated by Moraes et al., in their U2OS intracellular assay strain Y cycled every 4 days and was the least sensitive to benznidazole and nifurtimox (together with strain Dm28c TcI) [51] [strain Y has previously been characterised as partially resistant [70]]. Furthermore, in Moraes et al. ERA clone 2 (TcIV) was partially resistant to CYP51 inhibition, not reaching 100% inhibition which we also observed, while 92–80 clone 2 (TcV) showed the highest level of resistance giving a maximum inhibition of only 27% against posaconazole [51]. Interestingly, not only is strain 92–80 clone 2 the same DTU as strain PAH179 but Moraes et al. state it grows very slowly. A difference between the studies is that we did not see the low maximum inhibition with CLBrenerLuc for posaconazole that was observed by Moraes et al. using wild type CLBrener.

A previous report indicates that endogenous sterols need to be depleted before cell death occurs [42]. Our finding that posaconazole has very little effect on non-replicative trypomastigotes or that it is less active against more slowly replicating / cycling *T*. *cruzi* strains supports this conclusion. This suggests that several replications are required to see an effect in the presence of CYP51 inhibition. This is further supported by the EdU labelling experiment which shows that the fraction of actively replicating amastigotes in strain PAH179 is not changed by posaconazole treatment. Thus, replication per se does not provide sensitivity to CYP51 inhibition, instead a number of rounds of replication are required to see a cytocidal effect. However, it is possible that slow replication/ cycling correlates to another genetic factor which is directly involved in drug sensitivity / resistance.

A key result presented here is that long-term *in vitro* washout experiments, with the fast-replicating strain Silvio X10/7, show very clear differentiation between the nitroheterocyclic drugs and posaconazole. In particular, we demonstrate that even 16 days treatment with posaconazole at 50 fold the EC_50_ cannot prevent parasite recrudescence, whereas benznidazole treatment is able to prevent parasite recrudescence. Cal et al. [71] have previously carried out washout experiments with the same compounds but the conclusions from their study are limited by the short treatment duration used (4 days versus 16 days here) and short washout period (7 days versus 60 days here). The authors show that 4 days treatment with posaconazole does not kill all amastigotes, as could be expected from its slow rate-of-kill [53] and due to the relatively short washout period they could not unequivocally demonstrate complete killing of all parasites with benznidazole. Another key finding we present is that benznidazole does not prevent relapse after 8 days treatment, which is interesting as nearly all parasites are killed within 96 h of treatment [51, 53, 71]. Dormant parasites that are less susceptible to benznidazole *in vitro* have recently been reported [72] and our data supports the existence of such a subpopulation of persister parasites. Sánchez-Valdéz et al. further demonstrated that the relapsed parasites have not acquired permanent resistance to benznidazole. Importantly, the long interval between benznidazole washout and relapse (≥2 weeks) demonstrates the need for long-term *in vitro* washout assays to assess compounds and potentially the need for an extensive assessment period after immunosuppression in the *in vivo* model if sterile cure is to be demonstrated. Our findings correlate well with results from mouse model studies where posaconazole is not curative [52] and benznidazole treatment generally gives a much higher cure rate after 20 days compared to 10 days treatment [44, 73, 74].

Taken together we present three distinct *in vitro* assays where benznidazole performs well and posaconazole fails: the slow replicating / cycling strain potency assay, the trypomastigote assay, and the extended duration washout assay. These important differences offer a likely explanation for the poor performance of the CYP51 inhibitors E1224 and posaconazole in clinical trials [45–47, 75].

**Fig 5:**
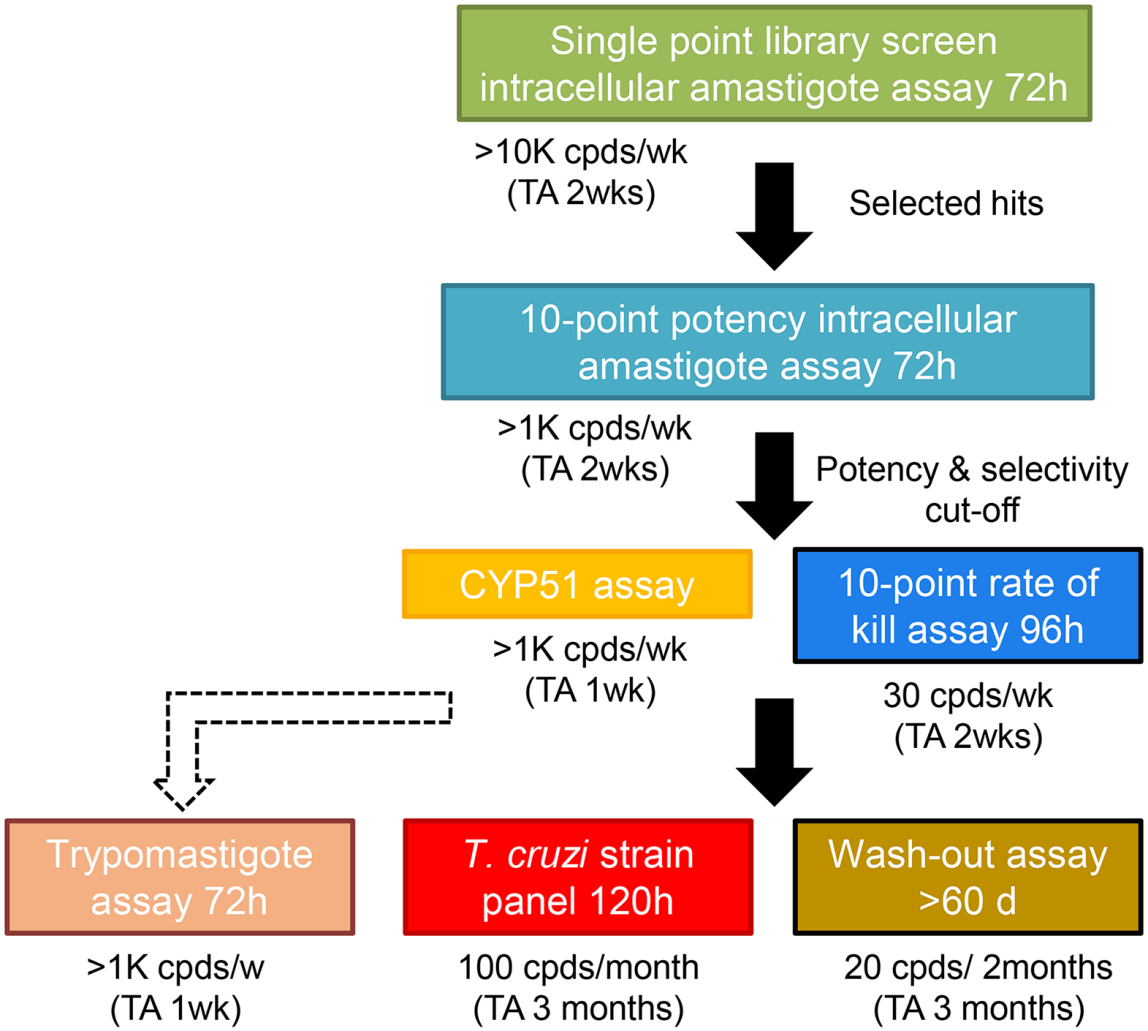
Updated *in vitro* screening cascade for Chagas’ disease with addition of the newly developed *T*. *cruzi* strain panel and washout assays. (Cpd, compound; TA, turnaround time).

We have incorporated these findings into our *in vitro* screening cascade for Chagas’ disease to improve translation. Hits are now selected from our 72 h single point intracellular assay and progressed to a cidal potency assay, these are then assessed for CYP51 mode of action. We now move representatives of non-CYP51 inhibitor series to a 5 day *T*. *cruzi* strain panel assay and the washout assay (>60 days). We also run compounds in our trypomastigote assay to determine efficacy against non-replicating stage parasites but, as is it not yet clear if trypomastigote killing is required for *in vivo* efficacy, this assay is not on the critical path of our *in vitro* screening cascade (Fig 5). While the inclusion of the *T*. *cruzi* strain panel allows us to assess a substantial level of genetic diversity by including one representative from each DTU, it is impossible to cover all *T*. *cruzi* variability, including intra-lineage variability.

Ultimately drugs will not only have to kill all disease-causing developmental forms, but also have the right properties to reach all the parasite reservoirs. This may be very challenging for a single non-reactive compound and combination therapy to address these issues may be the most likely route to success. As the current Chagas’ pipeline is very sparse there is a clear need for new anti-trypanosomal compounds with diverse modes of action.

## Supporting information

S1 Fig Triple assay genotyping profiles for *T*. *cruzi* strains. Silvio X10/7 (TcI), CLBrener (TcVI) & Tula (TcVI) Lanes 2, 3 and 4 respectively identified by PCR product size polymorphism of the *24S rRNA* locus **(A)**; PCR-RFLP based on SNP’s in *GPI* locus **(B)** and *HSP60* locus **(C).** DNA ladders (Bioline) Lane 1 Hyperladder V **(A)**, Lane 1 Hyperladder IV **(B & C).**
(TIFF)

S2 Fig Representative growth curves for all *T*. *cruzi* panel strains over 5 days. 32 technical replicates (average ±SD), 1 biological replicate.
(TIFF)

S3 Fig Raw dose-response data for a panel of *T*. *cruzi* strains after 5 days treatment with nifurtimox (A) benznidazole (B) and posaconazole (C). (3 biological replicates, dashed line curves for Tula and PAH179 refer to vertical axis on right). Silvio X10/7 and PAH179 plots are reproduced from Fig 2.
(TIF)

S4 Fig Detection of cells in S phase growth by EdU labelling of *L*. *donovani* LV9 mid-log promastigotes and Silvio X10/7 trypomastigotes. Cells labelled with EdU AlexFluor 488, Hoechst and anti-PFR1 antibody detected with goat anti-rabbit IgG AlexaFluor 647 (**A & C** respectively). Cells also stained with Hoechst and goat anti-rabbit IgG AlexaFluor 647 secondary antibody only as labelling controls (**B & D**). Bar 10 μm.
(TIFF)

S5 Fig EdU labelling of intracellular Silvio X10/7. Infected Vero treated for 5 days with DMSO **(A)** 5 μM nifurtimox **(B)** 50 μM benznidazole **(C)** and 1 μM posaconazole **(D)** labelled with EdU AlexaFluor 488 and Hoechst. Parasites remaining after treatment are highlighted by white arrows. Bar 20 μm.
(TIFF)

S6 Fig EdU labelling of intracellular PAH179. Infected Vero treated for 5 days with DMSO **(A)** 5 μM nifurtimox **(B)** 50 μM benznidazole **(C)** and 1 μM posaconazole **(D)** labelled with EdU AlexaFluor 488 and Hoechst. Parasites remaining after treatment are highlighted by white arrows. Bar 20 μm.
(TIFF)

S7 Fig Control for EdU labelling of intracellular Silvio X10/7. Infected Vero treated for 5 days with DMSO **(A)** 5 μM nifurtimox **(B)** 50 μM benznidazole **(C)** and 1 μM posaconazole **(D)** labelled with Hoechst only. Parasites remaining after treatment are highlighted by white arrows. Bar 20 μm.
(TIFF)

S8 Fig Control for EdU labelling of intracellular PAH179. Infected Vero treated for 5 days with DMSO **(A)** 5 μM nifurtimox **(B)** 50 μM benznidazole **(C)** and 1 μM posaconazole **(D)** labelled with Hoechst only. Parasites remaining after treatment are highlighted by white arrows. Bar 20 μm.
(TIFF)

S9 Fig Average percent EdU positive Vero cells (±SD) after 5 days treatment with DMSO, 5 μM nifurtimox, 50 μM benznidazole and 1 μM posaconazole. (TIFF)

S1 Table Key discrete typing unit (DTU) discriminating SNP’s in *T*. *cruzi TcSC5D* gene adapted from [64]. (DOCX)

S2 Table Summary of *T*. *cruzi* genotype profiles using a single locus assay discriminating key SNP’s in *T*. *cruzi TcSC5D* gene and a triple loci assay *(24S* PCR and PCR-RFLP *GPI* & *HSP60*). (DOCX)

S3 Table Drug potency & efficacy against *T*. *cruzi* panel strains at 72 h. pEC_50_ = –Log (EC_50_ [M]), average of at least three biological replicates ± SD. * 2/3 replicates pEC_50_ <4.3.
(DOCX)

S4 Table Drug potency & efficacy against *T*. *cruzi* panel strains at 96 h. pEC_50_ = –Log (EC_50_ [M]), average of at least three biological replicates ± SD.
(DOCX)

S5 Table Efficacy of nifurtimox, benznidazole and posaconazole against *T*. *cruzi* Silvio X10/7 and Tulahuen βgal strain trypomastigotes at 24 & 48 h. pEC_50_ = –Log (EC_50_ [M]), average of at least three biological replicates ± SD. * 2/3 replicates pEC_50_ <4.3.
(DOCX)
