# Supplementary figures and images for: Development of Trypanosoma cruzi in vitro assays to identify compounds suitable for progression in Chagas’ disease drug discovery
Source: PLoS Negl Trop Dis. 2018 Jul 12;12(7):e0006612. doi: 10.1371/journal.pntd.0006612 (PMC6057682; doi:10.1371/journal.pntd.0006612)

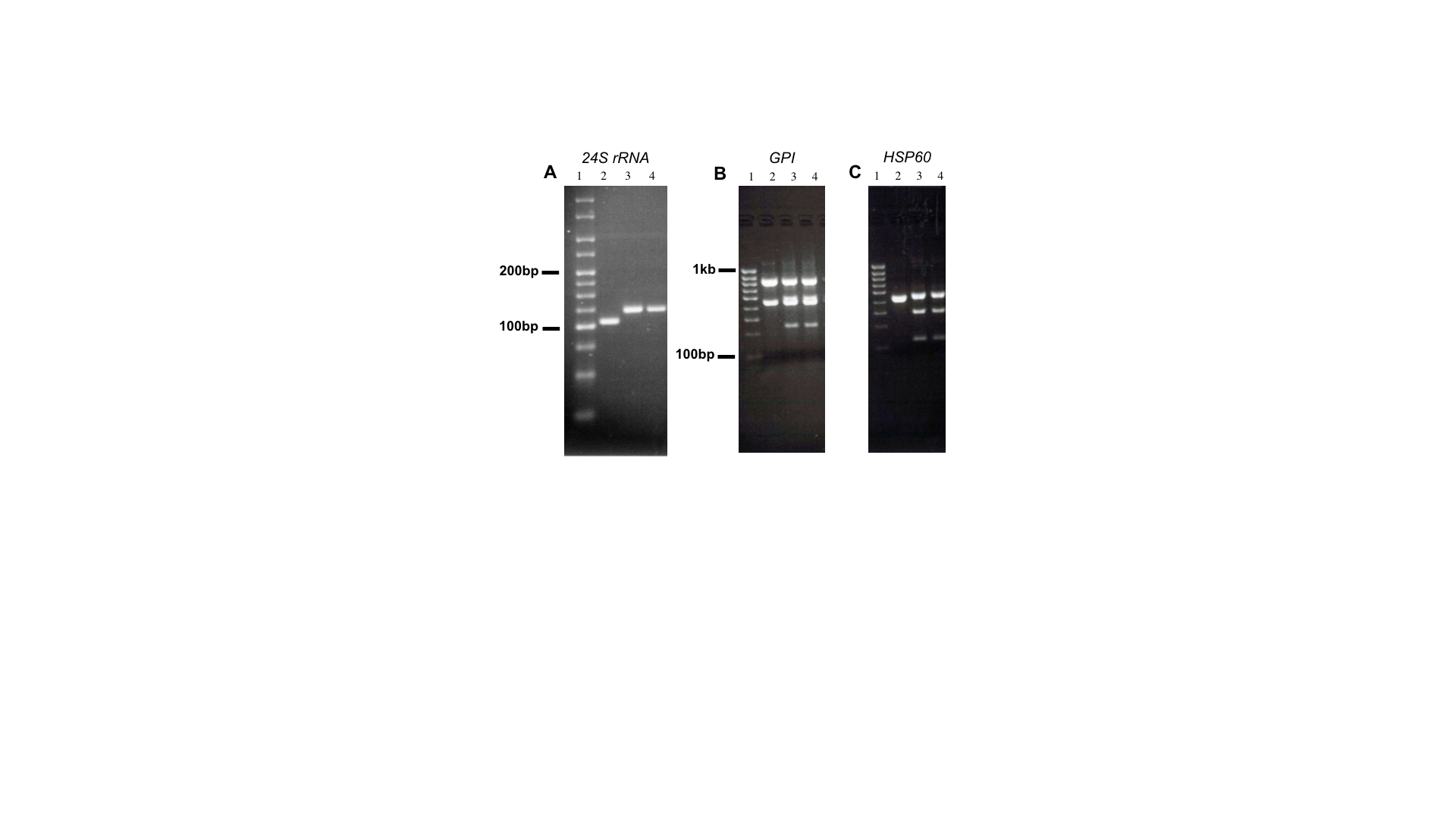

Supplement: S1 Fig — Silvio X10/7 (TcI), CLBrener (TcVI) & Tula (TcVI) Lanes 2, 3 and 4 respectively identified by PCR product size polymorphism of the 24S rRNA locus (A); PCR-RFLP based on SNP’s in GPI locus (B) and HSP60 locus (C). DNA ladders (Bioline) Lane 1 Hyperladder V (A), Lane 1 Hyperladder IV (B & C). (TIFF) [file pntd.0006612.s001.tiff]

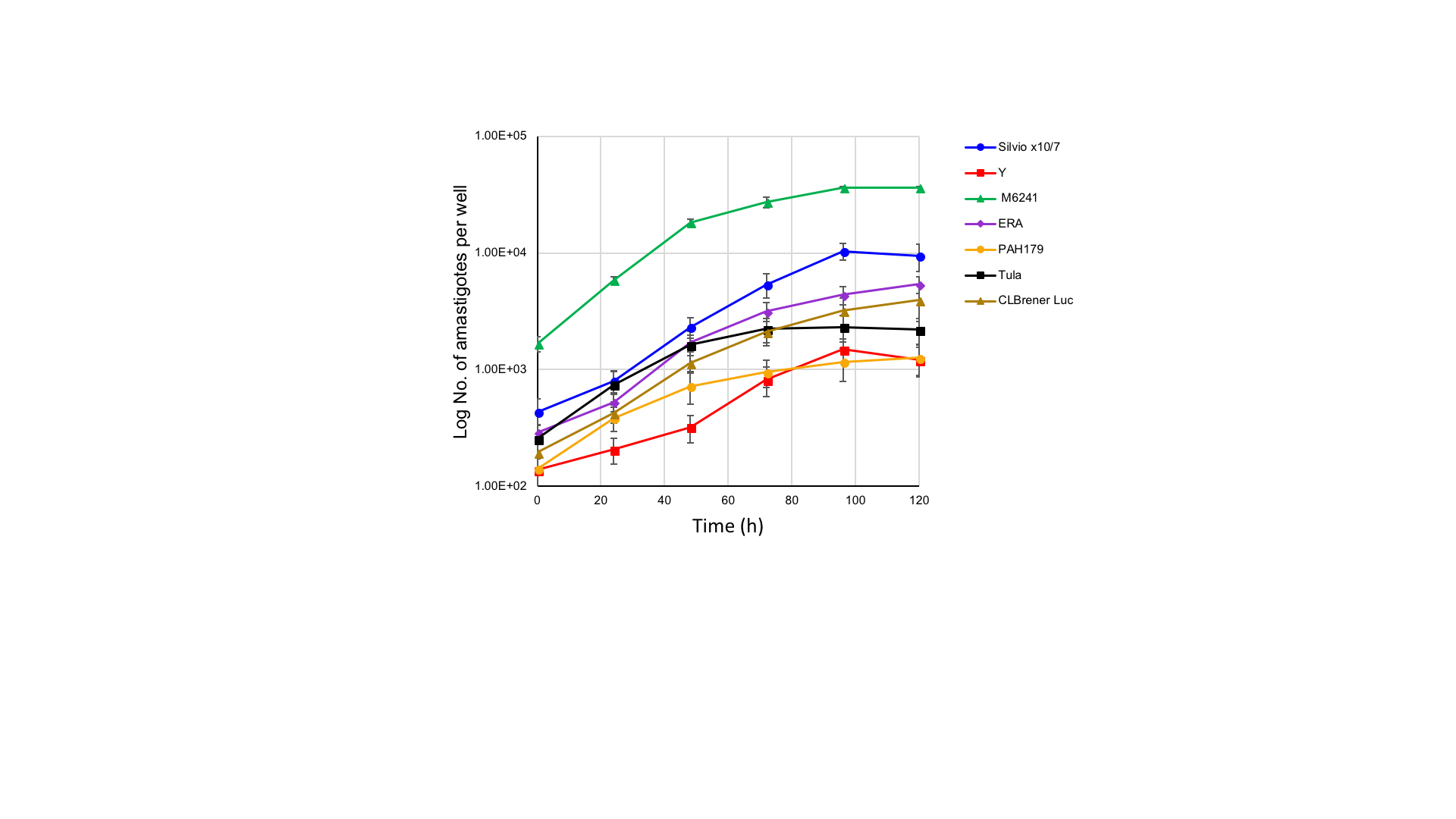

Supplement: S2 Fig — 32 technical replicates (average ±SD), 1 biological replicate. (TIFF) [file pntd.0006612.s002.tiff]

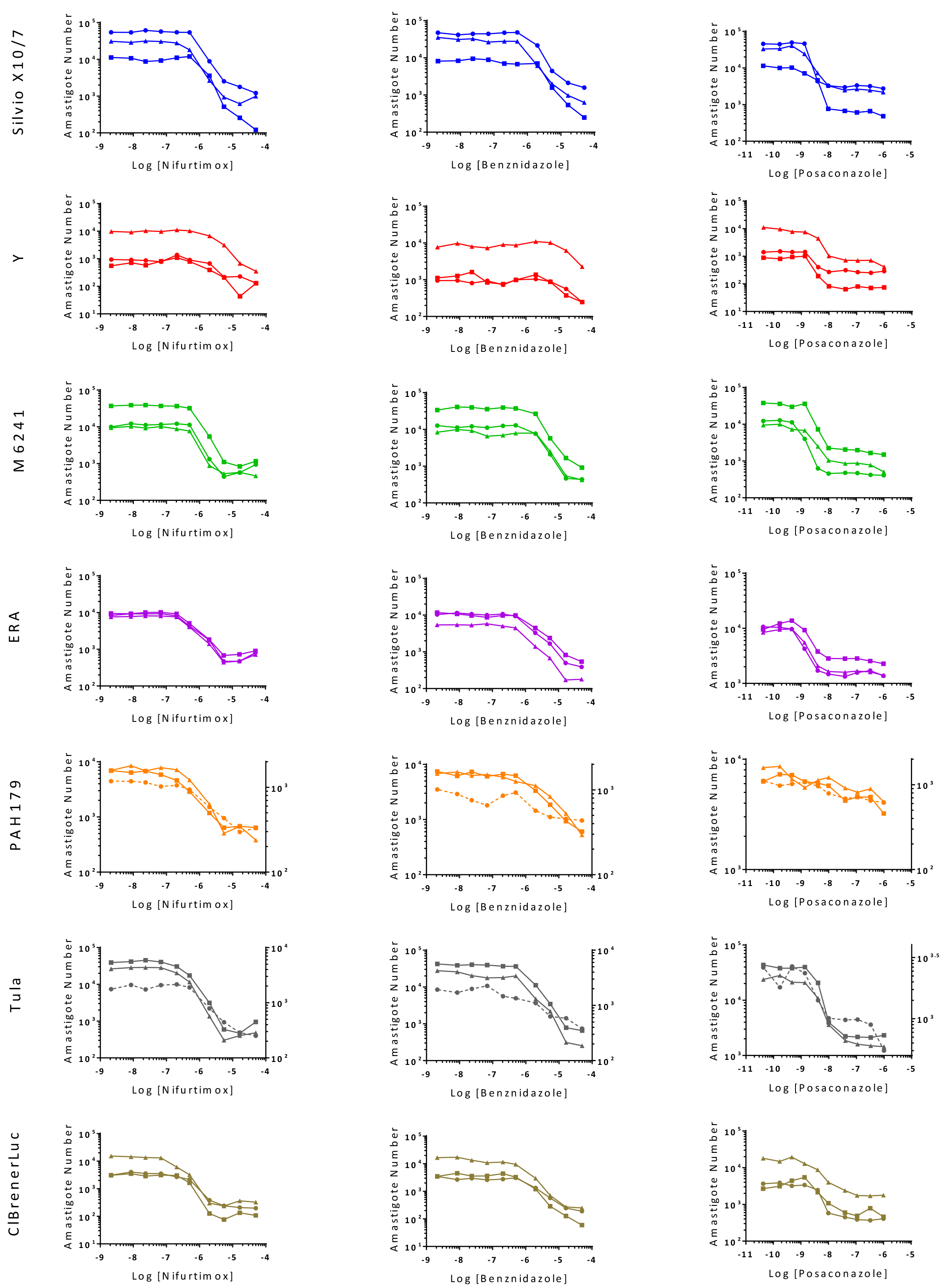

Supplement: S3 Fig — (3 biological replicates, dashed line curves for Tula and PAH179 refer to vertical axis on right). Silvio X10/7 and PAH179 plots are reproduced from Fig 2. (TIF) [file pntd.0006612.s003.tif]

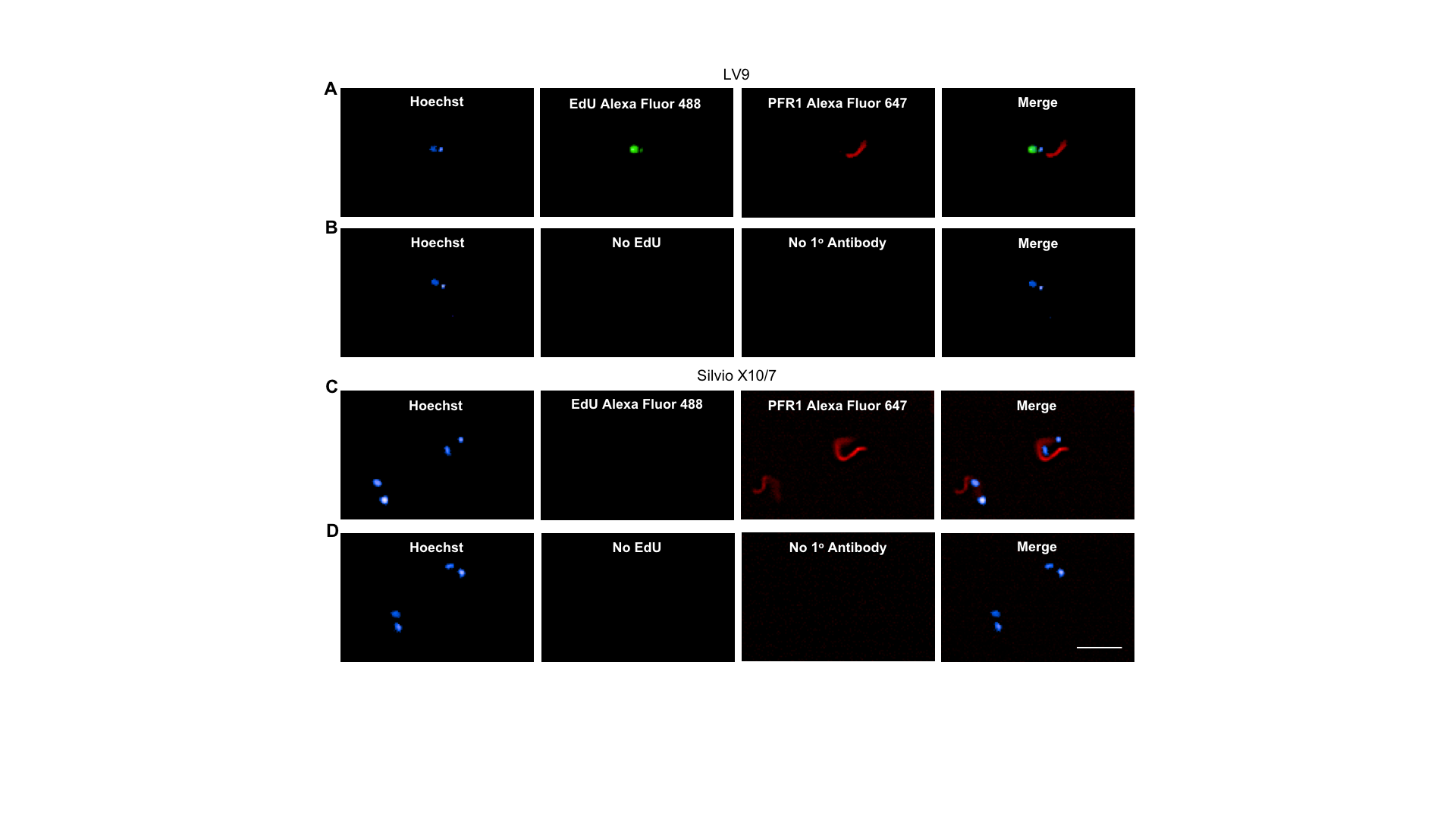

Supplement: S4 Fig — Cells labelled with EdU AlexFluor 488, Hoechst and anti-PFR1 antibody detected with goat anti-rabbit IgG AlexaFluor 647 (A & C respectively). Cells also stained with Hoechst and goat anti-rabbit IgG AlexaFluor 647 secondary antibody only as labelling controls (B & D). Bar 10 μm. (TIFF) [file pntd.0006612.s004.tiff]

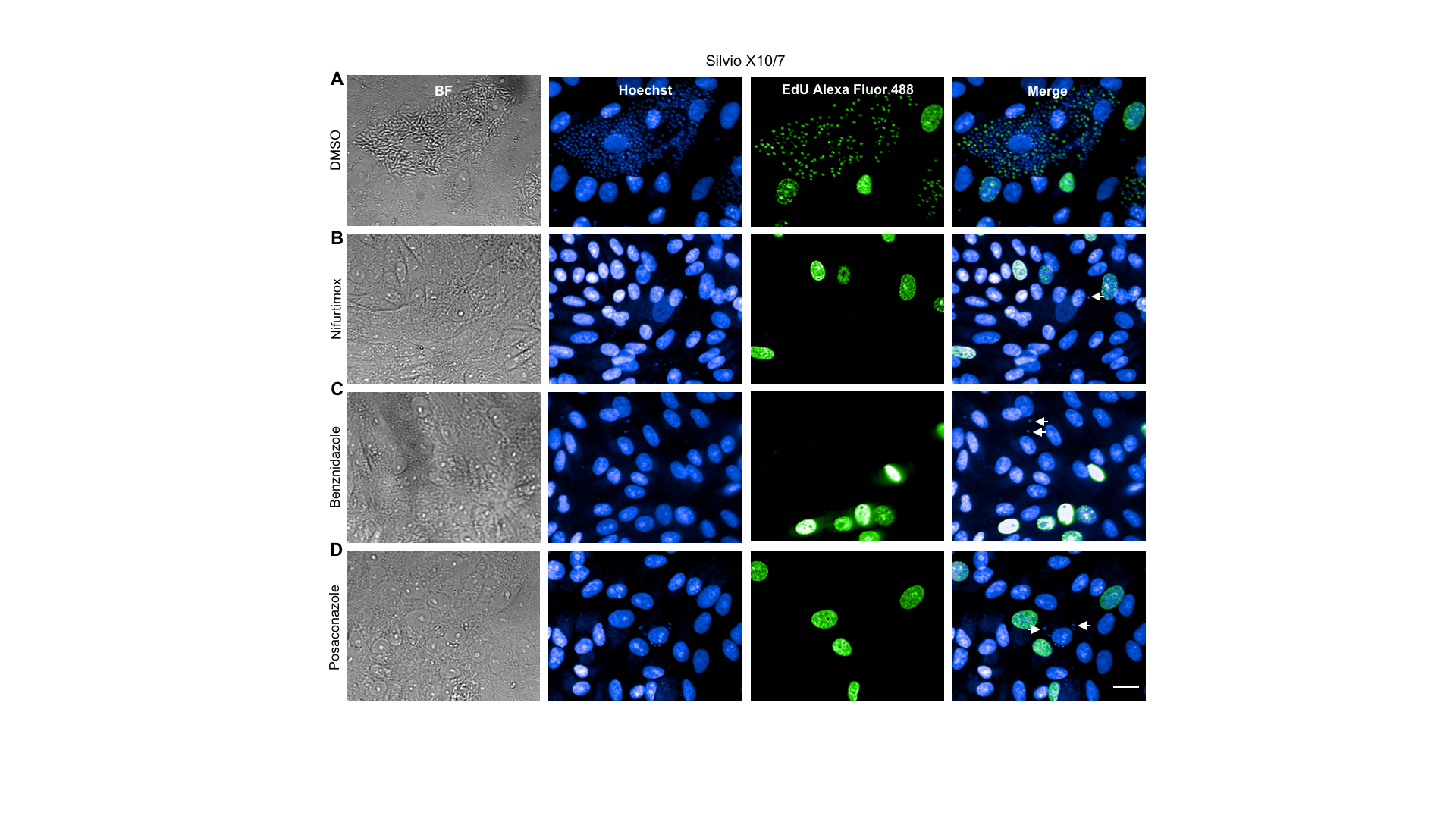

Supplement: S5 Fig — Infected Vero treated for 5 days with DMSO (A) 5 μM nifurtimox (B) 50 μM benznidazole (C) and 1 μM posaconazole (D) labelled with EdU AlexaFluor 488 and Hoechst. Parasites remaining after treatment are highlighted by white arrows. Bar 20 μm. (TIFF) [file pntd.0006612.s005.tiff]

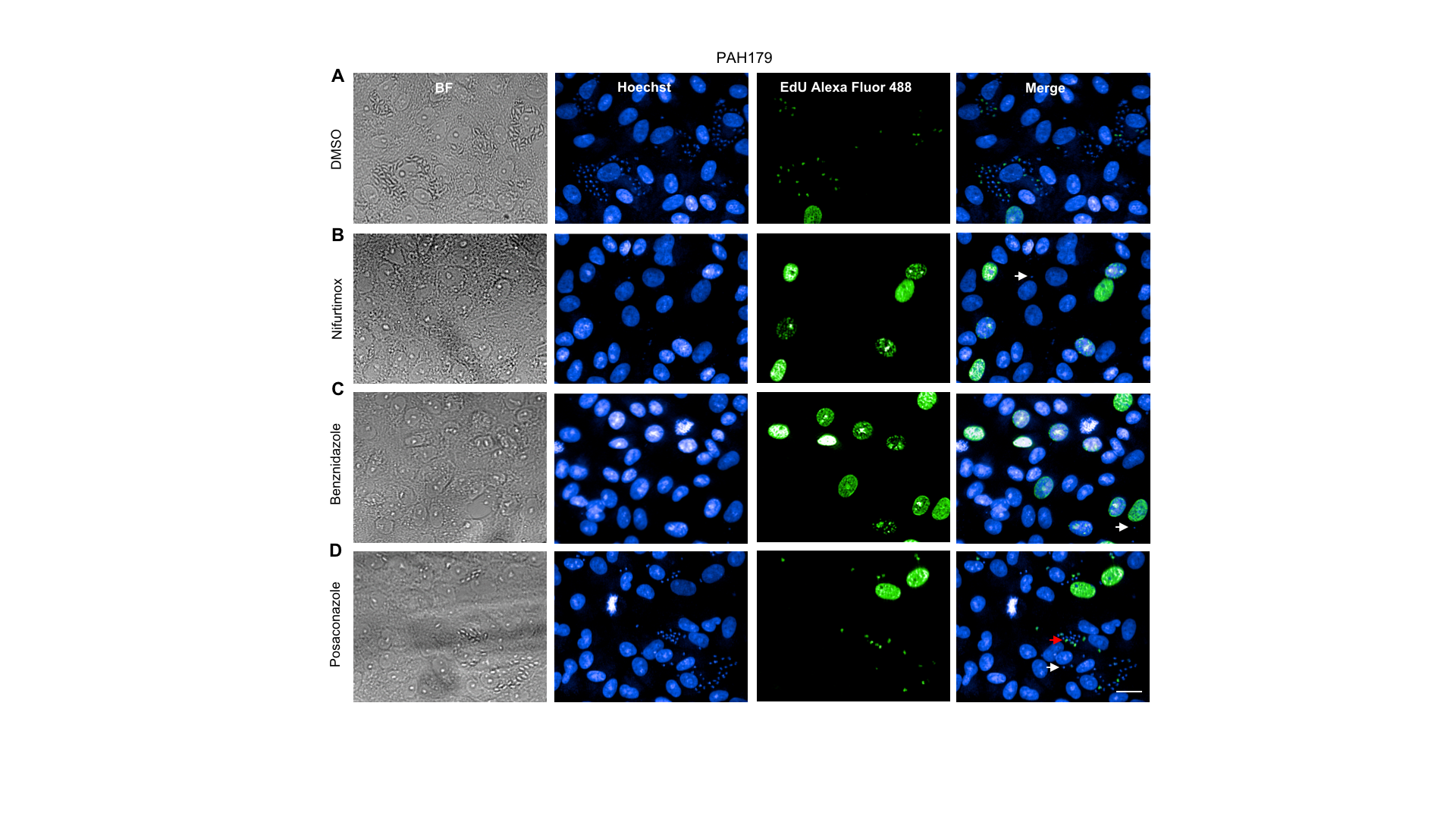

Supplement: S6 Fig — Infected Vero treated for 5 days with DMSO (A) 5 μM nifurtimox (B) 50 μM benznidazole (C) and 1 μM posaconazole (D) labelled with EdU AlexaFluor 488 and Hoechst. Parasites remaining after treatment are highlighted by white arrows. Bar 20 μm. (TIFF) [file pntd.0006612.s006.tiff]

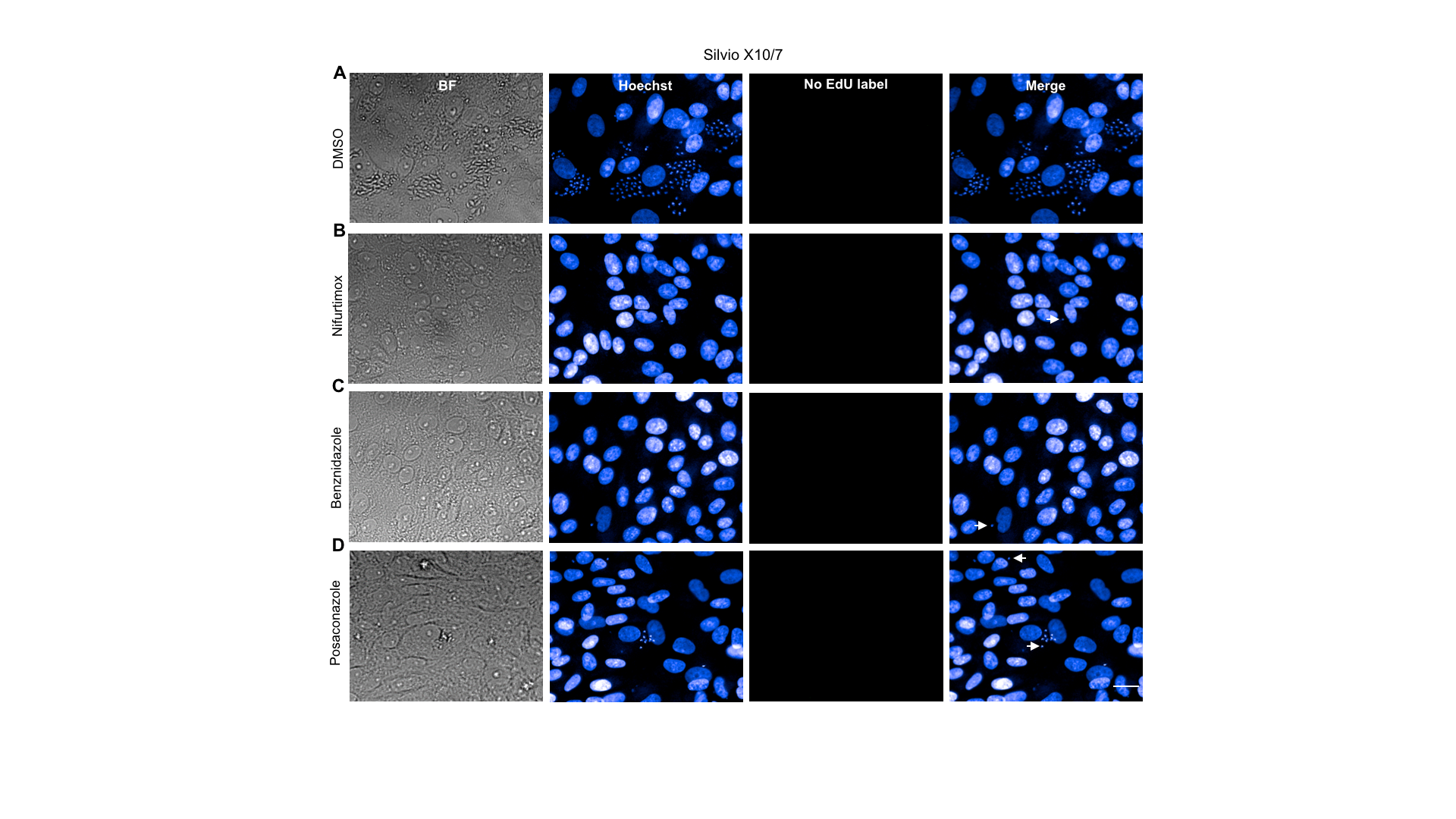

Supplement: S7 Fig — Infected Vero treated for 5 days with DMSO (A) 5 μM nifurtimox (B) 50 μM benznidazole (C) and 1 μM posaconazole (D) labelled with Hoechst only. Parasites remaining after treatment are highlighted by white arrows. Bar 20 μm. (TIFF) [file pntd.0006612.s007.tiff]

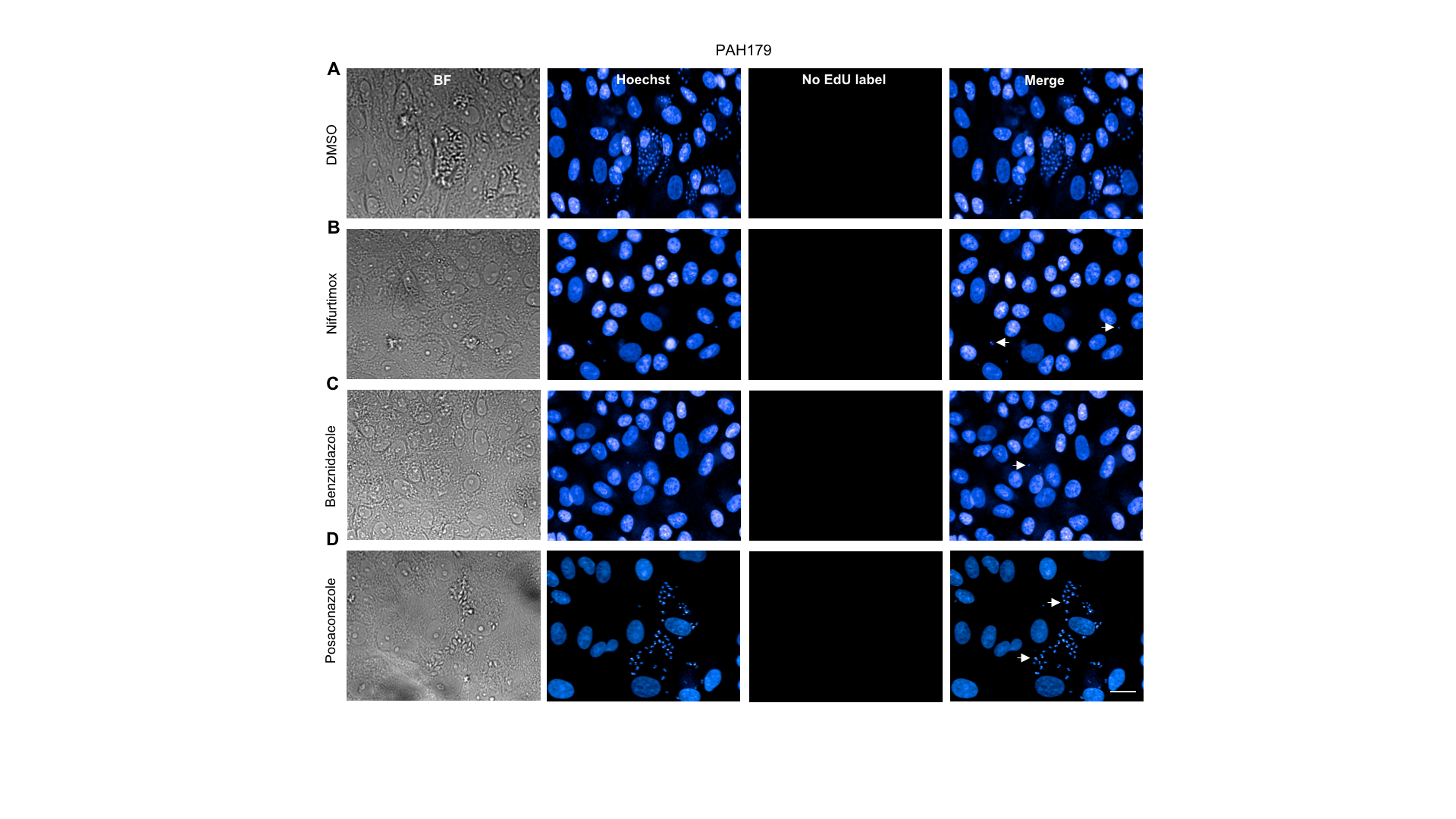

Supplement: S8 Fig — Infected Vero treated for 5 days with DMSO (A) 5 μM nifurtimox (B) 50 μM benznidazole (C) and 1 μM posaconazole (D) labelled with Hoechst only. Parasites remaining after treatment are highlighted by white arrows. Bar 20 μm. (TIFF) [file pntd.0006612.s008.tiff]

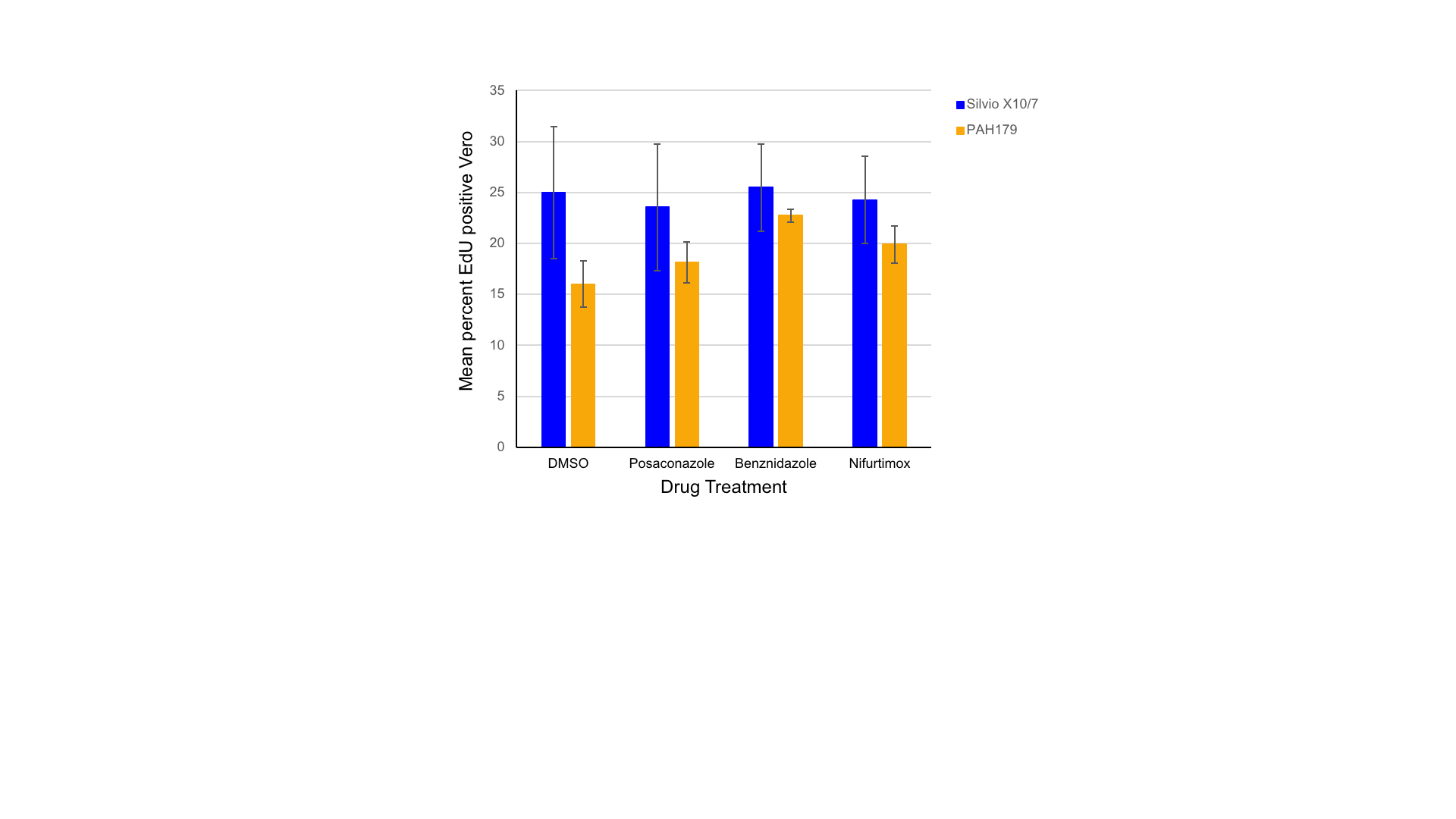

Supplement: S9 Fig — (TIFF) [file pntd.0006612.s009.tiff]
