# Supplementary material for: Development of Trypanosoma cruzi in vitro assays to identify compounds suitable for progression in Chagas’ disease drug discovery
Source: PLoS Negl Trop Dis. 2018 Jul 12;12(7):e0006612. doi: 10.1371/journal.pntd.0006612 (PMC6057682; doi:10.1371/journal.pntd.0006612)
Supplement: S1 Table — (DOCX) [file pntd.0006612.s010.docx]

**Supplementary Table 1.** Key DTU discriminating SNP’s in *T. cruzi* *TcSC5D* gene adapted from ([64](#_ENREF_64)).

|  | **SNP position *TcSC5D* gene** | | | | | | | |
| --- | --- | --- | --- | --- | --- | --- | --- | --- |
| **DTU** | 138 | 168 | 336 | 495 | 618 | 648 | 657 | 747 |
| **Tc I** | A | T | C | T | C | G | T | G |
| **Tc II** | T | G | T | T | C, T or C/T | T | G | T |
| **Tc III** | G | C | C | G | C | G | C | A |
| **Tc IV** | T | C | A | T | T | T | A | A |
| **Tc V** | G/T | C/G | C/T | G/T | C/T | G/T | C/G | A/T |
| **Tc VI** | G/T | C/G | C/T | G/T | C | G/T | C/G | A/T |
