# Supplementary material for: Development of Trypanosoma cruzi in vitro assays to identify compounds suitable for progression in Chagas’ disease drug discovery
Source: PLoS Negl Trop Dis. 2018 Jul 12;12(7):e0006612. doi: 10.1371/journal.pntd.0006612 (PMC6057682; doi:10.1371/journal.pntd.0006612)
Supplement: S2 Table — (DOCX) [file pntd.0006612.s011.docx]

**Supplementary Table 2.** Summary of *T. cruzi* genotype profiles using single locus assay discriminating key SNP’s in *T. cruzi* *TcSC5D* gene and a triple loci assay *(24S* PCR and PCR-RFLP *GPI* & *HSP60*).

|  | **SNP position *TcSC5D* gene** | | | | | | | | **Single Assay Genotype** | **Triple Assay Genotype** |
| --- | --- | --- | --- | --- | --- | --- | --- | --- | --- | --- |
| ***T.cruzi* Strain** | 138 | 168 | 336 | 495 | 618 | 648 | 657 | 747 |  |  |
| **Silvio X10/7 Clone A1** |  |  |  |  |  |  |  |  |  | **TcI** |
| **Y** | T | G | T | T | C/T | T | G | T | **TcII** | **TcII** |
| **M6241 Clone 6** | G | C | C | G | C | G | C | A | **TcIII** | **TcIII** |
| **ERA Clone 2** | T | C | A | T | T | T | A | A | **TcIV** |  |
| **PAH179 Clone 5** | G/T | C/G | C/T | G/T | C/T | G/T | C/G | A/T | **TcV** |  |
| **Tula Clone 2** |  |  |  |  |  |  |  |  |  | **TcVI** |
| **Tulahuen βgal** |  |  |  |  |  |  |  |  |  | **TcVI** |
