# Supplementary material for: Development of Trypanosoma cruzi in vitro assays to identify compounds suitable for progression in Chagas’ disease drug discovery
Source: PLoS Negl Trop Dis. 2018 Jul 12;12(7):e0006612. doi: 10.1371/journal.pntd.0006612 (PMC6057682; doi:10.1371/journal.pntd.0006612)
Supplement: S3 Table — pEC50 = –Log (EC50 [M]), average of at least three biological replicates ± SD. * 2/3 replicates pEC50 <4.3. (DOCX) [file pntd.0006612.s012.docx]

**Supplementary Table 3.** Drug potency & efficacy against *T. cruzi* panel strains at 72 h. pEC_50_ = –Log (EC_50_ [M]), average of at least three biological replicates ± SD. * 2/3 replicates pEC_50_ <4.3.

| ***T. cruzi* Strain ID** | **Nifurtimox** | | **Benznidazole** | | **Posaconazole** | | |
| --- | --- | --- | --- | --- | --- | --- | --- |
|  | **pEC_50_** | **Max inhibition (%)** | **pEC_50_** | **Max inhibition (%)** | | **pEC_50_** | **Max inhibition (%)** |
| Silvio X10/7 | 6.0 ± 0.2 | 101 ± 1 | 5.8 ± 0.1 | 100 ± 2 | | 8.2 ± 0.8 | 91 ± 8 |
| Y | 5.6 ± 0.2 | 108 ± 14 | 4.8* | 83 ± 8 | | 8.6 ± 0.3 | 100 ± 15 |
| M6241 | 6.2 ± 0.1 | 105 ± 3 | 5.6 ± 0.1 | 105 ± 3 | | 8.3 ± 0.6 | 98 ± 7 |
| ERA | 6.1 ± 0.1 | 102 ± 4 | 5.6 ± 0.2 | 102 ± 4 | | 8.8 ± 0.2 | 73 ± 15 |
| PAH179 | 5.9 ± 0.1 | 97 ± 4 | 5.4 ± 0.3 | 98 ± 3 | | <6.0 | 17 ± 9 |
| Tula | 6.2 ± 0.1 | 100 ± 0 | 5.6 ± 0.1 | 99 ± 1 | | 8.1 ± 0.6 | 67 ±8 |
| CLBrener Luc | 6.3 ± 0.2 | 100 ± 0 | 5.8 ± 0.1 | 100 ± 1 | | 8.3 ± 0.6 | 63 ± 15 |
