# Supplementary material for: Development of Trypanosoma cruzi in vitro assays to identify compounds suitable for progression in Chagas’ disease drug discovery
Source: PLoS Negl Trop Dis. 2018 Jul 12;12(7):e0006612. doi: 10.1371/journal.pntd.0006612 (PMC6057682; doi:10.1371/journal.pntd.0006612)
Supplement: S4 Table — pEC50 = –Log (EC50 [M]), average of at least three biological replicates ± SD. (DOCX) [file pntd.0006612.s013.docx]

**Supplementary Table 4.** Drug potency & efficacy against *T. cruzi* panel strains at 96 h. pEC_50_ = –Log (EC_50_ [M]), average of at least three biological replicates ± SD.

| ***T. cruzi* Strain ID** | **Nifurtimox** | | **Benznidazole** | | **Posaconazole** | | |
| --- | --- | --- | --- | --- | --- | --- | --- |
|  | **pEC_50_** | **Max inhibition (%)** | **pEC_50_** | **Max inhibition (%)** | | **pEC_50_** | **Max inhibition (%)** |
| Silvio X10/7 | 6.1 ± 0.2 | 100 ± 0 | 5.9 ± 0.1 | 100 ± 1 | | 8.6 ± 0.1 | 95 ± 3 |
| Y | 5.8 ± 0.3 | 105 ± 7 | 5.0 ± 0.1 | 88 ± 5 | | 8.6 ± 0.3 | 106 ± 8 |
| M6241 | 6.2 ± 0.1 | 104 ± 2 | 5.7 ± 0.1 | 104 ± 2 | | 8.7 ± 0.3 | 102 ± 3 |
| ERA | 6.2 ± 0.1 | 102 ± 2 | 5.9 ± 0.0 | 102 ± 2 | | 8.8 ± 0.2 | 87 ± 7 |
| PAH179 | 6.1 ± 0.3 | 101 ± 1 | 5.7 ± 0.3 | 100 ± 3 | | <6.0 | 25 ± 9 |
| Tula | 6.2 ± 0.3 | 98 ± 1 | 5.8 ± 0.1 | 96 ± 6 | | 8.4 ± 0.1 | 85 ± 6 |
| CLBrener Luc | 6.5 ± 0.3 | 100 ± 1 | 5.9 ± 0.3 | 100 ± 1 | | 8.2 ± 0.1 | 87 ± 5 |
