# Supplementary material for: Development of Trypanosoma cruzi in vitro assays to identify compounds suitable for progression in Chagas’ disease drug discovery
Source: PLoS Negl Trop Dis. 2018 Jul 12;12(7):e0006612. doi: 10.1371/journal.pntd.0006612 (PMC6057682; doi:10.1371/journal.pntd.0006612)
Supplement: S5 Table — pEC50 = –Log (EC50 [M]), average of at least three biological replicates ± SD. * 2/3 replicates pEC50 <4.3. (DOCX) [file pntd.0006612.s014.docx]

**Supplementary Table 5.** Efficacy of nifurtimox, benznidazole and posaconazole against *T. cruzi* Silvio X10/7 and Tulahuen βgal strain trypomastigotes at 24 & 48 h. pEC_50_ = –Log (EC_50_ [M]), average of at least three biological replicates ± SD. * 2/3 replicates pEC_50_ <4.3.

| ***T. cruzi* Strain ID** |  | **Nifurtimox** | | **Benznidazole** | | | **Posaconazole** | |
| --- | --- | --- | --- | --- | --- | --- | --- | --- |
|  |  | **pEC_50_** | **Max inhibition (%)** | **pEC_50_** | **Max inhibition (%)** | **pEC_50_** | | **Max inhibition (%)** |
| Silvio X10/7 | 24 | 5.2 ± 0.0 | 100 ± 0 | 4.8 ± 0.0 | 92 ± 2 | <4.3 | | 37 ± 2 |
|  | 48 | 5.6 ± 0.0 | 100 ± 0 | 5.1 ± 0.0 | 99 ± 1 | 5.1* | | 41 ± 13 |
| Tulahuen βgal | 24 | 5.4 ± 0.1 | 100 ± 1 | 4.6 ± 0.1 | 93 ± 1 | <4.3 | | 18 ± 15 |
|  | 48 | 5.9 ± 0.1 | 101 ± 1 | 5.0 ± 0.0 | 100 ± 1 | <4.3 | | 4 ± 15 |
